# Long-Term Exposure to Source-Specific Fine Particles and Mortality—A Pooled Analysis of 14 European Cohorts within the ELAPSE Project

**DOI:** 10.1021/acs.est.2c01912

**Published:** 2022-06-23

**Authors:** Jie Chen, Gerard Hoek, Kees de Hoogh, Sophia Rodopoulou, Zorana J. Andersen, Tom Bellander, Jørgen Brandt, Daniela Fecht, Francesco Forastiere, John Gulliver, Ole Hertel, Barbara Hoffmann, Ulla Arthur Hvidtfeldt, W. M. Monique Verschuren, Karl-Heinz Jöckel, Jeanette T. Jørgensen, Klea Katsouyanni, Matthias Ketzel, Diego Yacamán Méndez, Karin Leander, Shuo Liu, Petter Ljungman, Elodie Faure, Patrik K. E. Magnusson, Gabriele Nagel, Göran Pershagen, Annette Peters, Ole Raaschou-Nielsen, Debora Rizzuto, Evangelia Samoli, Yvonne T. van der Schouw, Sara Schramm, Gianluca Severi, Massimo Stafoggia, Maciej Strak, Mette Sørensen, Anne Tjønneland, Gudrun Weinmayr, Kathrin Wolf, Emanuel Zitt, Bert Brunekreef, George D. Thurston

**Affiliations:** † Institute for Risk Assessment Sciences (IRAS), Utrecht University, 3584 CM Utrecht, The Netherlands; ‡ Swiss Tropical and Public Health Institute, 4051 Basel, Switzerland; § University of Basel, 4001 Basel, Switzerland; ∥ Department of Hygiene, Epidemiology and Medical Statistics, Medical School, National and Kapodistrian University of Athens, 115 27 Athens, Greece; ⊥ Section of Environment and Health, Department of Public Health, University of Copenhagen, 1165 Copenhagen, Denmark; # Institute of Environmental Medicine, Karolinska Institutet, SE-171 77 Stockholm, Sweden; ¶ Centre for Occupational and Environmental Medicine, Region Stockholm, 113 65 Stockholm, Sweden; ∇ Department of Environmental Science, Aarhus University, Frederiksborgvej 399, DK-4000 Roskilde, Denmark; ○ iClimate—Interdisciplinary Center for Climate Change, Aarhus University, Frederiksborgvej 399, DK-4000 Roskilde, Denmark; ⧫ MRC Centre for Environment and Health, School of Public Health, Imperial College London, Norfolk Place, W2 1PG London, U.K.; †† Department of Epidemiology, Lazio Region Health Service, ASL Roma 1, 00147 Rome, Italy; ‡‡ Environmental Research Group, School of Public Health, Imperial College London, W2 1PG London, U.K.; §§ Centre for Environmental Health and Sustainability & School of Geography, Geology and the Environment, University of Leicester, LE1 7RH Leicester, U.K.; ∥∥ Department of Ecoscience, Aarhus University, 4000 Roskilde, Denmark; ⊥⊥ Institute for Occupational, Social and Environmental Medicine, Centre for Health and Society, Medical Faculty, Heinrich Heine University Düsseldorf, 40001 Düsseldorf, Germany; ## Danish Cancer Society Research Center, 2100 Copenhagen, Denmark; ¶¶ National Institute for Public Health and the Environment, 3720 BA Bilthoven, The Netherlands; ∇∇ Julius Center for Health Sciences and Primary Care, University Medical Center Utrecht, Utrecht University, 3584 CG Utrecht, the Netherlands; ○○ Institute for Medical Informatics, Biometry and Epidemiology, Medical Faculty, University of Duisburg-Essen, 45259 Essen, Germany; ⧫⧫ Global Centre for Clean Air Research (GCARE), University of Surrey, GU2 7XH Guildford, United Kingdom; ††† Department of Global Public Health, Karolinska Institutet, 171 77 Stockholm, Sweden; ‡‡‡ Centre for Epidemiology and Community Medicine, Region Stockholm, 113 65 Stockholm, Sweden; §§§ Department of Cardiology, Danderyd University Hospital, 182 88 Stockholm, Sweden; ∥∥∥ University Paris-Saclay, UVSQ, Inserm, Gustave Roussy, “Exposome and Heredity” Team, CESP UMR1018, 94805 Villejuif, France; ⊥⊥⊥ Department of Medical Epidemiology and Biostatistics, Karolinska Institutet, 171 77 Stockholm, Sweden; ### Institute of Epidemiology and Medical Biometry, Ulm University, Helmholtzstrasse 22, 89081 Ulm, Germany; ¶¶¶ Institute of Epidemiology, Helmholtz Zentrum München, 85764 Neuherberg, Germany; ∇∇∇ Chair of Epidemiology, Ludwig Maximilians Universität München, 81377 Munich, Germany; ○○○ Department of Neurobiology, Care Sciences, and Society, Karolinska Institutet and Stockholm University, 171 77 Stockholm, Sweden; ⧫⧫⧫ Department of Statistics, Computer Science and Applications “G. Parenti” (DISIA), University of Florence, 50121 Firenze FI, Italy; †††† Department of Natural Science and Environment, Roskilde University, 4000 Roskilde, Denmark; ‡‡‡‡ Agency for Preventive and Social Medicine (aks), 6900 Bregenz, Austria; §§§§ Department of Internal Medicine 3, LKH Feldkirch, 6800 Feldkirch, Austria; ∥∥∥∥ Departments of Environmental Medicine and Population Health, New York University Grossman School of Medicine, New York, 10010-2598 New York, United States

**Keywords:** source apportionment, fine particulate matter (PM_2.5_), absolute principal component analysis (APCA), mortality

## Abstract

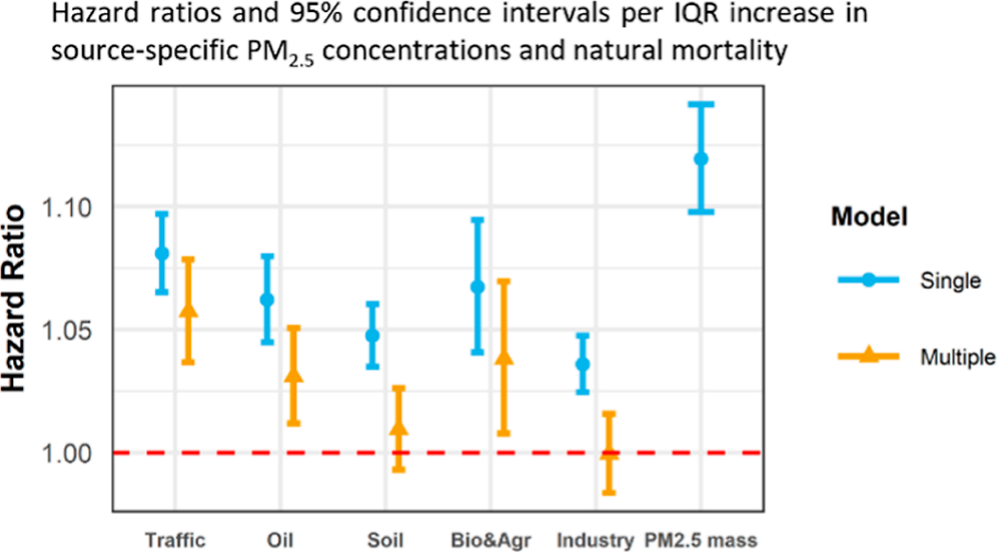

We assessed mortality risks associated with source-specific fine particles (PM_2.5_) in a pooled European cohort of 323,782 participants. Cox proportional hazard models were applied to estimate mortality hazard ratios (HRs) for source-specific PM_2.5_ identified through a source apportionment analysis. Exposure to 2010 annual average concentrations of source-specific PM_2.5_ components was assessed at baseline residential addresses. The source apportionment resulted in the identification of five sources: traffic, residual oil combustion, soil, biomass and agriculture, and industry. In single-source analysis, all identified sources were significantly positively associated with increased natural mortality risks. In multisource analysis, associations with all sources attenuated but remained statistically significant with traffic, oil, and biomass and agriculture. The highest association per interquartile increase was observed for the traffic component (HR: 1.06; 95% CI: 1.04 and 1.08 per 2.86 μg/m^3^ increase) across five identified sources. On a 1 μg/m^3^ basis, the residual oil-related PM_2.5_ had the strongest association (HR: 1.13; 95% CI: 1.05 and 1.22), which was substantially higher than that for generic PM_2.5_ mass, suggesting that past estimates using the generic PM_2.5_ exposure response function have underestimated the potential clean air health benefits of reducing fossil-fuel combustion. Source-specific associations with cause-specific mortality were in general consistent with findings of natural mortality.

## Abstract 2

Understanding particulate air pollution from which sources are of greater health impact than others would inform targeted policy controls. This study reports that residual fuel oil-related particles are most strongly associated with mortality risks.

## 1 Introduction

Epidemiological studies around the world have generally reported associations between fine particle mass (PM_2.5_) exposure and mortality and morbidity, with variations in the magnitude of effect estimates.^1^ Part of these effect size fluctuations per unit mass may be related to the fact that the composition of PM_2.5_ mass varies in time and space, depending on sources of emission and atmospheric chemistry, which may in turn result in differences in toxicity and risk to health of PM_2.5_ mass.^2−5^ Understanding which components of the PM mixture are of greater health impact than others would help inform targeted policies to control PM_2.5_ from those sources that contribute most of the toxic components in the PM mixture as well as allow more accurate assessments of source-specific health impacts.

To date, many studies have reported associations between adverse health outcomes and long-term exposure to a series of PM_2.5_ constituents, including secondary inorganic aerosols (sulfate, nitrate), black carbon (BC), metals, and organic components, with no consistent evidence of a single constituent being most strongly related to health effects.^6−12^ In our previous analyses within the Effects of Low-level Air Pollution: A Study in Europe (ELAPSE), we found that vanadium (V) within PM_2.5_ was most consistently associated with increased mortality risks in the pooled cohort of 323,782 participants from six European countries,^13^ whereas potassium (K) and silicon (Si) were most robustly associated with natural mortality in almost 27 million participants from six large administrative cohorts.^14^ However, analyses on a constituent basis may be difficult to interpret because individual elements can be linked to one or more specific sources (e.g., differing by location-specific source mixtures) and thus have different associated health effects, depending on what aerosol mixture they travel with (i.e., which source group they are in), while several covarying elemental markers may more reliably indicate the same source.^15−17^ For example, iron (Fe) may come from wind-blown soil, a steel mill operation, or brake wear, but it is associated with different elements, depending on which source (e.g., with Si for soil, Mn for steel, and Cu for brake wear).^18^ Looking at Fe in a source-specific tracer group can differentiate the mixture situations. This factor may help explain uncertainty as to which source/constituent is more strongly related to health effects in past PM_2.5_ constituent evaluations (e.g., US EPA, 2021).^19^

Observed health associations with individual PM_2.5_ trace elements are not necessarily causal but may rather indicate associations with the source-specific mixture they are part of. Furthermore, results from a model which includes multiple elements emitted from the same source (e.g., V and Ni from residual oil combustion) are difficult to interpret, as the model could bias the effect estimates for individual elements.^20,21^ To avoid this possibility, assessing health effects of PM components as a group (e.g., source-specific groupings of tracers) may provide more consistent and interpretable results across studies than trying to parse effects among individual elements. Source-specific associations are also more readily translatable into air quality policy than elemental associations. For example, an analysis within the American Cancer Society Cancer Prevention Study II (ACS CPS-II) suggested exposure to coal combustion-related air pollution explained most associations between PM_2.5_ mass and increased risk of mortality from all causes, ischemic heart diseases (IHDs), and lung cancer (LC) in the US.^22,23^ In the California Teachers study, associations with IHD mortality were observed for sources including gasoline- and diesel-fueled vehicles, meat cooking, and high-sulfur fuel combustion.^6^ The National Particle Component Toxicity (NPACT) studies identified that secondary sulfate and traffic sources were most consistently associated with adverse health effects.^22,24^

In the present study, we performed a further analysis within the ELAPSE pooled cohort to consider the mortality risks associated with long-term PM_2.5_ exposure on a source-specific basis. By comparing these source-specific results with our previous individual elemental analyses, we expected to have a more complete understanding of the health effects of PM_2.5_ mixtures from different sources.

## 2 Materials and Methods

### 2.1 Study Population

The ELAPSE pooled cohort consists of 14 subcohorts across six European countries. Detailed information on individual subcohorts has been extensively reported.^13,25,26^ The included subcohorts are: the Cardiovascular Effects of Air Pollution and Noise in Stockholm (CEANS) cohort in Sweden, which in turn includes the Stockholm Diabetes Prevention Program (SDPP),^27^ the Stockholm Cohort of 60-year-olds (SIXTY),^28^ Stockholm Screening Across the Lifespan Twin study (SALT),^29^ and Swedish National Study on Aging and Care in Kungsholmen (SNAC-K);^30^ the Diet, Cancer, and Health cohort (DCH)^31^ in Denmark; the Danish Nurse Cohort (DNC)^32^ in Denmark, consisting at baseline of two surveys conducted in 1993 and 1999; the European Prospective Investigation into Cancer and Nutrition-Netherlands (EPIC-NL) cohort in the Netherlands, including the Monitoring Project on Risk Factors and Chronic Diseases in the Netherlands (MORGEN) and Prospect;^33^ the Heinz Nixdorf Recall study (HNR) in Germany;^34^ the Etude Epidémiologique auprès de femmes de la Mutuelle Générale de l’Education Nationale (E3N) in France;^35^ the Cooperative Health Research in the Region of Augsburg (KORA) in Germany, consisting at baseline of two cross-sectional population-representative surveys conducted in 1994–1995 (S3) and 1999–2001 (S4); and the Vorarlberg Health Monitoring and Prevention Programme (VHM&PP) in Austria.^36^ Most cohorts covered a large city and its surrounding areas as study areas. The French E3N cohort and the Danish DNC cohort are national cohorts. All included cohort studies were approved by the medical ethics committees in their respective countries.

### 2.2 Source Apportionment and Exposure Assessment

Air pollution measurements for PM_2.5_ mass, NO_2_, BC, and PM_2.5_ elemental composition were derived from the ESCAPE monitoring campaign conducted in 19 study areas across Europe. Sampling and analysis methods have been described before.^16,37^ Briefly, measurements were made at 20 sites in each study area (40 in the large Catalunya and Netherlands/Belgium areas) for three 2 week periods in a 1 year period between October 2008 and April 2011. Monitoring sites were selected using a common sampling protocol to represent pollution levels at regional background, urban background, and street locations, with a focus on urban areas and in streets with only several regional background sites located outside of major urban areas.^16^ Eight components were a priori selected in the ESCAPE study: copper (Cu), Fe, K, nickel (Ni), sulfur (S), Si, V, and zinc (Zn).^16,38^ Annual average concentrations were calculated based on the measurements spread over the seasons (warm, cold, and intermediate) with temporal adjustment from a reference background site in each study area. Table D1, Supporting Information documents the distribution of air pollution measurements.

The 2010 annual average concentrations of air pollution from 397 sites were analyzed to estimate source apportioned PM_2.5_ mass exposures using absolute principal component analysis (APCA).^39^ The method involved the following: (a) applying PCA to the pollution data; (b) identifying source-related components based on key tracers in each component; (c) adjusting PC scores into absolute PC scores; and (d) regressing PM_2.5_ mass on the source-related components (i.e., the absolute PC scores), providing apportionments of PM_2.5_ mass to each identified source-related component. PM_2.5_ concentrations not attributable to the identified source components were incorporated into the model intercept. The APCA approach is further elaborated in Section A, Supporting Information. A five-factor PCA using Promax rotation (a type of oblique rotation)^40^ was chosen as the optimal solution. This decision was based on the goal to keep PCs having factor eigenvalues (i.e., the data variance explained by the identified component) greater than 1.0 after rotation, and the cumulative percentage of variance explained larger than 80%, as recommended by Hopke,^41^ as well as an examination of the source-related interpretability of the factors. We first tried a five-factor Varimax rotated approach (a type of orthogonal rotation) that resulted in 28.7% negative contributions estimated for the third component, indicating a non-optimal rotation of the PCs, which is likely due to the forcing of orthogonality (i.e., varimax rotation) when sources are actually correlated with each other in real world. We therefore instead applied an oblique rotation approach (i.e., Promax rotation) to allow the identified source components to be intercorrelated with one another, which is more realistic in real-world settings. The process to derive the optimal APCA solution is documented in Section B, Supporting Information.

Particulate K is an element enriched in biomass burning emissions but can also be in soil dust.^15^ In order to facilitate the interpretation of source-specific components, we attempted to obtain a more specific metric for K by looking at the non-soil K separately. We therefore adjusted the K concentrations with the tracer of crustal soil dust (i.e., Si) to exclude that K component before the source identification PCA. The adjusted K index was calculated by subtracting the soil dust-associated K from the total K concentration values (*K*_w_ = *K* – 0.42 × Si). The coefficient was calculated by regressing the K against Si concentrations for those samples with the lowest 10th percentile K/Si ratio samples.^42^ PM_2.5_ S was not included in the initial source identification analysis because S is considered as a general marker for fossil fuel sources and may complicate the separation of fine mass to specific sources.^43^ Also, excluding tracers of secondary formation (i.e., S) from the source apportionment analysis allows a clearer discrimination of the original primary sources of PM_2.5_.^44^ S was then apportioned among the sources by regressing it against the identified sources to calculate the “unexplained” secondary mass prior to the PM_2.5_ mass apportionment regression models.^44^ Based on the APCA, source-specific compositional profiles were assessed by regressing each pollutant against the mass contributions for all sources together in a linear model. Source profiles of S were similarly assessed, though S was not included in the PCA.

To evaluate the robustness of our source apportionment to the approach chosen, we performed sensitivity analyses, including: (1) fivefold robustness evaluation; (2) adding S into the source identification PCA; and (3) including total K concentrations in the PCA (i.e., without removing the dust soil-associated K component). In the fivefold robustness evaluation, the full set of measurements was randomly divided into five groups (20% each), stratified by site type (street, rural, and urban background) and region (north, west, central, and south). Five additional APCA analyses were performed, each based on a randomly selected 80% of the monitoring sites. We note that there is 60% overlap between any two training sets of the sites.

The APCA results were then applied to exposure estimates of NO_2_, BC, and individual elemental components to assess source-specific PM_2.5_ exposures. This involved first converting individual exposures to the absolute PC scores for all identified source components and then multiplied by the regression slopes derived from the PM_2.5_ mass measurement apportionment. Individual exposure to 2010 annual mean concentrations of PM_2.5_ mass, NO_2_, BC, and PM_2.5_ elemental composition was assessed at participants’ baseline residential addresses based on Europe-wide land use regression model estimates.^45,46^ The models were previously built on ground-based measurements with satellite-derived and chemical transport modeled air pollutant estimates, land use, road, and population data as predictors. The models explained a moderate to large fraction of the measured concentration variation at the European scale (i.e., 66% for PM_2.5_, 58% for NO_2_, 51% for BC, and 41 to 79% across elemental components).

### 2.3 Mortality Outcome Definition

Identification of outcomes was based upon linkage to mortality registries within each cohort. Based on the underlying cause of death recorded on death certificates according to the International Classification of Diseases, Ninth Revision (ICD-9)^47^ and the International Statistical Classification of Diseases and Related Health Problems, Tenth Revision (ICD-10),^48^ we defined mortality from natural causes (ICD-9: 001-779, ICD-10: A00-R99), cardiovascular diseases (CVDs) (ICD-9: 400–440, ICD-10: I10–I70), non-malignant respiratory diseases (ICD-9: 460–519, ICD-10: J00–J99), and LC (ICD-9: 162, ICD-10: C34).

### 2.4 Statistical Analyses

Cox proportional hazard regression models were applied to evaluate associations of identified PM_2.5_ sources with natural and cause-specific mortality, following the general ELAPSE analytical framework applied in our previous paper of elemental exposures.^13,26,49^ Death from other causes, emigration, loss to follow-up for other reasons, or withdrawn alive at the end of follow-up were considered censoring events. The Cox models were stratified by subcohorts to account for differences in baseline hazards between the subcohorts unexplained by the available covariates and to relax the proportional hazards assumption. The decision to account for between subcohort heterogeneity with strata implies that we mostly evaluate within-cohort exposure contrasts. Three confounder models were a priori specified with increasing adjustment for individual and area-level covariates: model 1 adjusted for age (as the time axis), subcohort (as strata), sex (as strata), and year of enrollment; model 2 further added individual-level covariates including marital status (married/cohabiting, divorced/separated, single, and widowed), smoking status (never, former, and current), smoking duration (years of smoking) for current smokers, smoking intensity (cigarettes/day) for current smokers, squared smoking intensity, body mass index (BMI) categories (<18.5, 18.5–24.9, 25–29.9, and >30 kg/m^2^), and employment status (employed vs unemployed); and model 3 further adjusted for area-level mean income in 2001. Model 3 was considered as the main model. Participants with missing exposure or incomplete information on model 3 covariates were excluded from all main analyses to allow comparison between models with increasing covariate control.

Individual PM_2.5_ sources were included as linear functions in the Cox models as a reasonable summary of the association as well as to facilitate comparisons with previous studies. Besides the single-source analysis (where one PM_2.5_ source was evaluated at a time), multisource analyses were also performed with all identified PM_2.5_ sources included in the model simultaneously for comparison. Cumulative risks of all identified sources were estimated assuming additive effects of combined source exposures on mortality. Cumulative risk index (CRI) was defined as
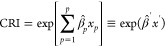
where β̂′ = (β̂_1_, ... β̂_*p*_) are the log-hazard ratio (HR) for the P source exposures estimated at *x*_*p*_ concentrations in a Cox model consisting of all P sources together.^50^ HRs and 95% confidence intervals (CIs) for interquartile range (IQR) increases in the estimated concentrations of each source were reported. In addition, HRs associated with per 1 μg/m^3^ increase are presented.

To evaluate the potential selection bias introduced by excluding participants with incomplete information on model 3 covariates, we compared model 1 HRs derived from analyses conducted in the model 3 population (i.e., with complete covariate information) and the model 1 population (i.e., with missing covariate information). To assess the impact of temporal misalignment of the exposure assessment, we performed sensitivity analyses starting the follow-up from 2000, 2005, 2008, and 2010, as per the previous analyses in the same cohort.^13^ To assess the uncertainty of source estimation effects on source-specific mortality results, we applied APCA results derived from the fivefold robustness evaluation analysis described above, in addition to the APCA results derived from the chosen optimal approach.

All analyses were performed in R version 3.4.0 using packages: *survival*, *coxme*, *Matrix*, *foreach*, *multcomp*, *survey*, *splines*, *Hmisc*, *mfp*, *VIM*, *ggplot2*, *frailtySurv*, *survsim*, *eha*, *stamod*, and *psych*. Statistical significance was based on a 95% CI of effect estimate not including unity.

## 3 Results and Discussion

### 3.1 Source Apportionment Results

**Figure 1:**
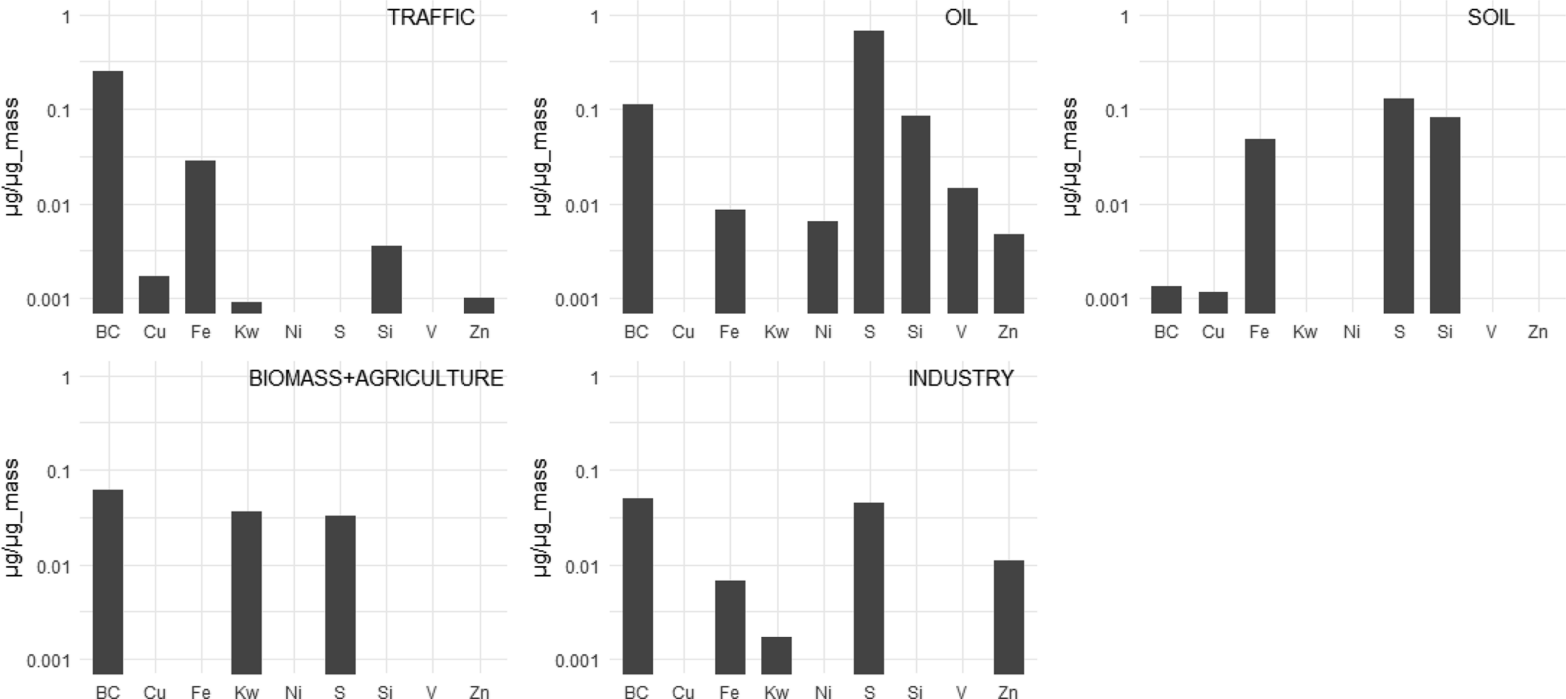
Estimated fractional elemental source profiles of identified source-specific PM_2.5_.

Table 1 provides the correlations between the identified source components and the individual air pollutants (i.e., factor loadings). PM_2.5_ mass and S were not included in the PCA, but their correlations with the identified factors were calculated to aid in the interpretation of the source components. Overall, the source apportionment resulted in a plausible identification of source contributions, with the possible exception of a biomass source, where we did not have access to a more specific marker, such as levoglucosan. The first factor was identified as traffic-related particles because of its high loadings on NO_2_, BC, Cu, and Fe. The principle sources of NO_2_ and BC include the combustion processes from motorized traffic and off-road machinery,^51,52^ whereas Cu and Fe are both considered as markers of brake wear.^15,16^ The interpretation of the first factor predominantly reflecting traffic is supported by the documentation of clearly higher concentrations of NO_2_, BC, Cu, and Fe at traffic locations compared to urban background locations of the monitoring database.^16,37^ The second factor was identified as particles from residual fuel oil combustion, based on its high loadings on both Ni and V, two elements known to be enriched in heavier fuel oils.^17^ Residual fuel oil, often burned by marine shipping, industry, and electric power plants, is the oil that remains after the removal of more valuable (and usually cleaner burning) distillates, such as gasoline, from petroleum. Ni and V have been mainly linked to shipping emissions in Europe.^17^ In the monitoring database, high concentrations of V and Ni were primarily measured in port cities including Rotterdam, Athens, Barcelona, and cities with significant industrial activities such as Turin and the German Ruhr area,^16^ suggesting marine shipping is likely a dominant source for this factor. The third factor was identifiable as crustal/soil particles because of its high loading on Si and moderate loading on Fe. Si is a specific tracer for the crustal material and Fe is also abundant in the crustal dust.^15^ The fourth component was first identified as particles from biomass burning because of its high loading on *K*_w_, which is the most common element used to trace biomass burning.^15^ The identification of the fourth factor is rather uncertain, however, because of the high explained variance by S in the source profile (Figure 1), and the fact that there is no S in wood. We speculated that this factor may possibly also include windblown soil containing agricultural fertilizers because both K and S are in fertilizers widely used in Europe.^53^ We therefore identified the fourth factor as particles from biomass and agriculture. The last factor was identified as associated with industrial emissions because of its high loading on Zn. Even though Zn was selected as a tracer for non-tailpipe traffic emitted particles in ESCAPE,^16^ it was not found to correlate with other traffic tracers in this analysis (e.g., NO_2_, BC, and Cu). However, Zn is also a tracer for industrial emitted particles, as also supported by our Europe-wide models, where predictors representing industrial emitted Zn explained a predominant part of the variation in Zn measurements.^45^ Correlations at the monitoring sites between identified source-specific PM_2.5_ were low to moderate (Table D2, Supporting Information).

**Table 1:** Factor Loadings for the Five-Factor Promax Rotated Principal Component Analysis Solution (Kappa = 1.6) [*S and PM_2.5_ Not Included in Factor Analysis]^a^

	traffic	oil	soil	biomass and agriculture	industry
NO_2_	**0.97**	0.10	–0.09	–0.11	0.00
BC	**0.88**	0.03	0.00	0.22	0.10
Cu	**0.86**	–0.02	0.22	0.06	0.04
Fe	**0.71**	0.02	0.46	–0.06	0.09
*K*_w_	0.02	–0.02	0.00	**0.99**	0.03
Ni	0.10	**0.91**	0.10	–0.01	0.03
Si	0.10	0.20	**0.89**	0.01	0.00
V	–0.01	**0.95**	0.06	–0.01	0.02
Zn	0.15	0.06	0.01	0.03	**0.93**
*S	–0.01	0.43	0.38	0.27	0.20
*PM_2.5_	0.44	0.04	0.17	0.46	0.25
eigenvalue	3.01	1.80	1.07	1.04	0.88
cumulative var	33.5%	53.4%	65.3%	76.9%	86.7%

a *K*_w_ is soil-adjusted K.

The source compositional profiles show that S explained a large fraction of all the identified source-related mass, except for traffic-related particles (Figure 1). Again, S was not included in the initial source identification analysis and was therefore distributed over the identified sources. Sulfate is a secondary pollutant formed through a series of atmospheric reactions. Its precursor sulfur dioxide (SO_2_) is emitted by the burning of liquid and solid fuels that contain S. The estimation of trace elements associated with each source component aids in the identification of the source and provides perspective on the mass estimates.

Sensitivity analyses supported our main source apportionment approach (Section C, Supporting Information). The five additional APCA each based on 80% of the monitoring sites showed similar results to the main analysis, that is the same sources with similar loadings of individual elements were found (Tables C1 and C2, Figure C1, Supporting Information), suggesting our main results are robust. Including S in the PCA resulted in similar separation of source components to the main PCA approach (Table C3 and Figure C2, Supporting Information). We expected including S in the PCA would undermine the clear separation of the fossil fuel combustion sources (e.g., residual oil and traffic on one component) because particulate S results from transformation of sulfur dioxide emitted from many fossil fuel combustion sources and thus it is not unique to any one source. It is reassuring to see similar separation of source components using both approaches and it can be considered more a philosophical choice whether to include S in the PCA analysis or not. Including total K without adjusting for soil dust-associated K resulted in similar factor loadings but higher contributions in soil component from K (Table C4 and Figure C3,Supporting Information).

### 3.2 Population Characteristics and Source-Specific PM_2.5_ Exposure Estimation

Of the total population of 381,036 study participants, 323,782 (85%) had complete information on model 3 covariates and were included in the main analyses (Table 2). The participants were followed up for an average of 19.5 years, contributing to 6,317,235 person-years of follow-up. Most of the study cohorts started from mid-1990 and were followed-up till 2011–2015. The average age at baseline ranged from 42 to 73. Four subcohorts included only female participants, and the pooled cohort comprised 66% females. Differences across the cohorts were also observed for the population size, average years of follow-up, socioeconomic status (SES), and lifestyle factors, supporting our decision to account for difference in baseline hazards between subcohorts. Detailed baseline characteristics of study population in individual subcohorts can be found elsewhere.^13,25,26^

**Table 2:** Population Characteristics

cohort	population size (*N*)^a^	population in main model 3 [*N* (%)]	baseline period	follow-up	average years of follow-up	age at baseline (mean ± SD)	female (%)	current smokers (%)	married or living with partner (%)	employed (%)	mean area-level income, *1000€ (mean ± SD)
pooled cohort	381,036	323,782 (85.0)			19.5	48.7 ± 13.4	66	24	72	70	20.1 ± 5.8
CEANS-SDPP	7835	7716 (98.5)	1992–1998	2011	15.9	47.1 ± 4.9	61	26	84	91	24.3 ± 4.2
CEANS-SIXTY	4180	3965 (94.9)	1997–1999	2014	15.5	60.0 ± 0.0	52	21	74	68	24.7 ± 6.9
CEANS-SALT	6724	6174 (91.8)	1998–2003	2011	10.4	57.8 ± 10.6	55	21	68	64	25.3 ± 6.6
CEANS-SNACK	3248	2830 (87.1)	2001–2004	2011	7.4	72.9 ± 10.4	62	14	46	23	28.7 ± 2.2
DCH	56,308	52,779 (93.7)	1993–1997	2015	18.2	56.7 ± 4.4	53	36	71	78	20.1 ± 3.4
DNC-1993	19,664	17,017 (86.5)	1993	2013	18.7	56.2 ± 8.4	100	37	68	70	19.2 ± 2.6
DNC-1999	8769	8117 (92.6)	1999	2013	14.4	47.9 ± 4.2	100	29	76	95	19.0 ± 2.4
EPIC_NL-Morgen	20,711	18,292 (88.3)	1993–1997	2013	16.8	42.9 ± 11.3	55	35	65	69	12.2 ± 1.6
EPIC_NL-Prospect	16,194	14,570 (90.0)	1993–1997	2013	16.4	57.7 ± 6.1	100	23	77	51	13.1 ± 1.4
HNR	4809	4733 (98.4)	2000–2003	2015	12	59.7 ± 7.8	50	24	75	40	25.2 ± 8.2
E3N	53,521	38,537 (72.0)	1989–1991	2011	16.8	53.0 ± 6.8	100	13	83	68	11.2 ± 3.0
KORA-S3	4566	2572 (56.3)	1994–1995	2011	15.6	49.4 ± 13.9	51	20	80	55	36.7 ± 4.4
KORA-S4	4257	2281 (53.6)	1999–2001	2014	12.9	49.3 ± 13.8	51	23	79	59	38.0 ± 7.3
VHM&PP	170,250	144,199 (84.7)	1985–2005	2014	23.1	42.1 ± 15.0	56	20	69	70	22.9 ± 1.7

a Population size is the number of subjects for which information was transferred to Utrecht University for construction of the pooled cohort.

**Figure 2:**
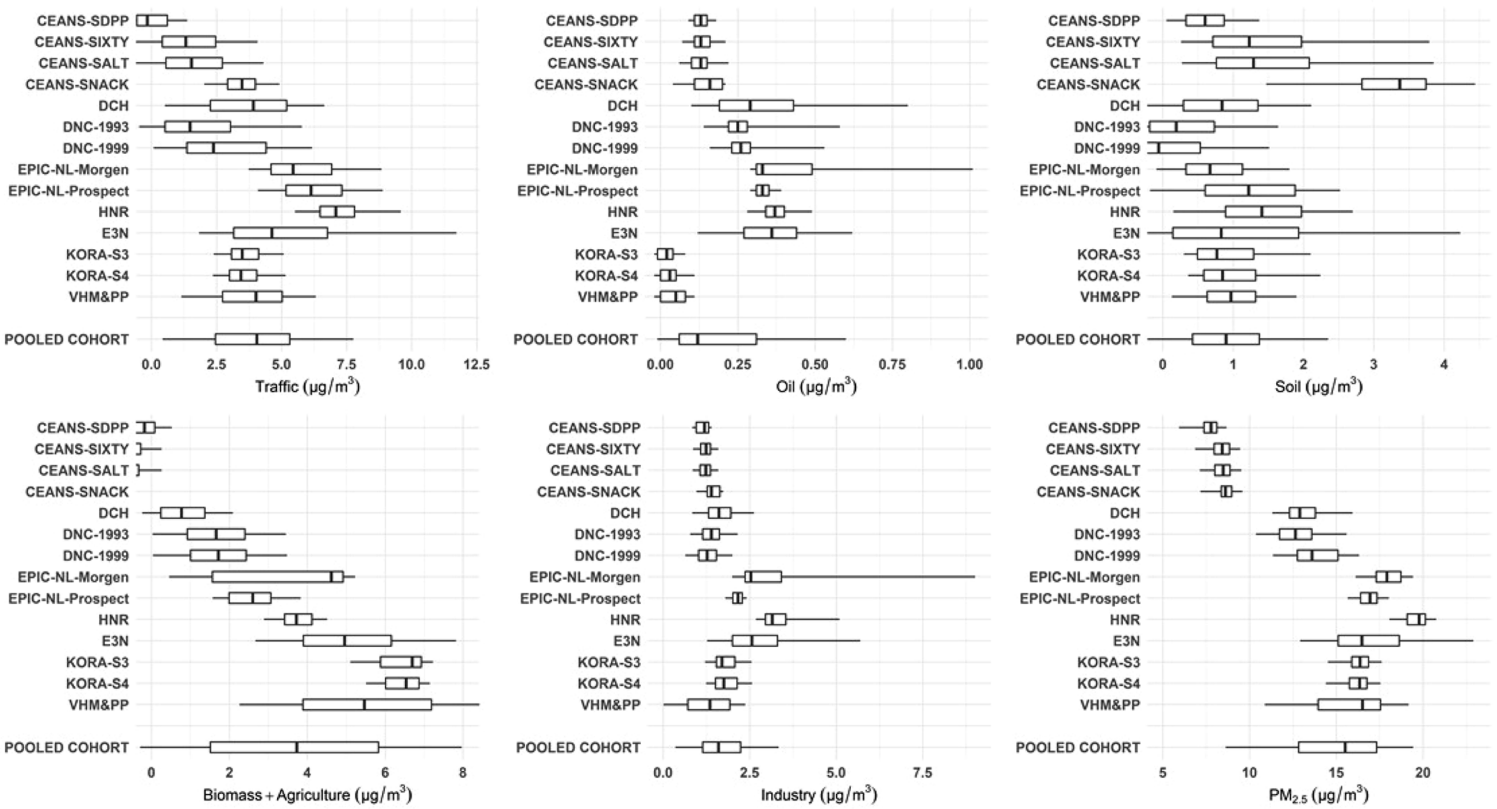
Exposure distribution of source-specific PM_2.5_ concentrations at participants’ baseline residential addresses. Subcohorts are shown from North to South; the boundary of the box closest to zero indicates P25; the boundary of the box furthest from zero, P75; the bold vertical line inside the box, P50; and the whiskers, P5 and P95. Exposure distribution for the pooled cohort is shown in Table D3,Supporting Information.

Figure 2 and Table D3,Supporting Information show the exposure distribution of the identified source-specific PM_2.5_ concentrations estimated for the study population in the individual subcohorts and the pooled cohort. Exposure concentrations generally showed a North–South increasing trend for PM_2.5_ from traffic and biomass and agriculture and the generic PM_2.5_ mass. The within-cohort exposure variability was large for PM_2.5_ from traffic, soil, and biomass and agriculture. PM_2.5_ exposures from residual oil burning and industrial sources were low in all subcohorts with relatively small within-cohort exposure contrasts, mainly because of the lack of sources in the study areas. An exception was observed for a small number of subjects within the Dutch EPIC-NL-Morgen cohort with high exposures in PM_2.5_ from both residual oil burning and industrial sources, likely related to shipping emissions from port cities and emissions from steel industries.^54^ Soil-related PM_2.5_ exposures ranged similarly across subcohorts, except the relatively high exposures observed for participants within the SNACK cohort located in Kungsholmen, Stockholm, which could be related to winter sanding of streets and road abrasion from studded tires. We estimated relatively low PM_2.5_ concentrations from soil, and relatively high PM_2.5_ concentrations from biomass and agriculture. This suggested that we may not be able to completely disentangle biomass burning-associated K from soil dust-associated K, even with the adjustment of K concentrations. However, the high estimate for biomass contribution was comparable with a PM_2.5_ source apportionment study previously conducted in Europe.^55^ Moreover, the windblown soil contribution to PM is predominately in the coarse fraction, and the traffic-associated soil mass from road dust may have been picked up by (i.e., attributed to) the traffic component and thus resulted in the low contribution of this non-traffic soil component.

Correlations between source-specific PM_2.5_ exposures at residential addresses were moderate to low (Table D4, Supporting Information), allowing proper interpretation of the multisource analyses. We reported the median of cohort-specific correlations, as it is most relevant for our interpretations because analyses were stratified by subcohort. Correlations between sources varied across subcohorts. The values were not directly comparable to the correlations between identified sources at the monitoring sites presented in Table D2, Supporting Information as the correlations at monitoring sites were assessed at the European scale.

### 3.3 Mortality Risks Associated with Source-Specific PM_2.5_

**Figure 3:**
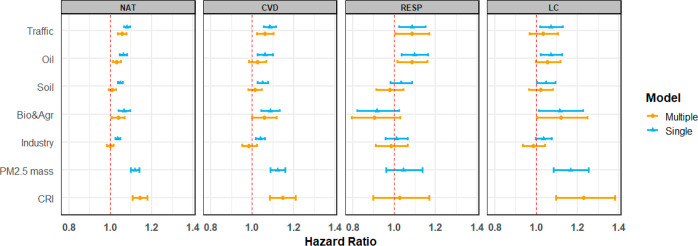
Associations of source-specific PM_2.5_ with mortality from natural-cause (NAT), CVDs, non-malignant respiratory diseases (RESP), and LC. HRs and 95% CIs are presented for the increment of the IQR for each exposure in the pooled cohort: traffic 2.86, μg/m^3^; oil, 0.25 μg/m^3^; soil, 0.95 μg/m^3^; biomass and agriculture, 4.32 μg/m^3^; industry, 1.09 μg/m^3^; and PM_2.5_ mass, 4.49 μg/m^3^ (Table D3,Supporting Information). See Table D5, Supporting Information for the corresponding numeric data. Total number of observations = 323,782; person-years at risk = 6,317,235; deaths from natural mortality = 46,640; deaths from cardiovascular mortality = 15,492; deaths from non-malignant respiratory mortality = 2846; and deaths from LC mortality = 3776. The main model adjusted for subcohort identification, age, sex, year of enrollment, smoking (status, duration, intensity, and intensity^2^), BMI categories, marital status, employment status, and 2001 area-level mean income.
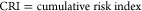

During the follow-up, we observed 46,640 (14.4%), 15,492 (4.8%), 2846 (0.9%), and 3776 (1.2%) deaths from natural causes, CVDs, non-malignant respiratory diseases, and LC respectively. Figure 3 and Table D5, Supporting Information show the associations between source-specific PM_2.5_ and natural and cause-specific mortality.

We observed significantly positive associations with traffic-related PM_2.5_ for all assessed mortality endpoints in single-source models. In multisource models, associations decreased slightly for mortality from natural causes, CVDs and LC, and remained stable for respiratory mortality with slightly wider CIs. The HRs associated with a per IQR increase in source-specific PM_2.5_ concentrations were the highest for the traffic component for all assessed endpoints except LC mortality (HR: 1.06; 95% CI: 1.04 and 1.08 for natural mortality per 2.86 μg/m^3^ increase in traffic-related PM_2.5_). Consistent findings were reported by the ACS CPS-II and the California Teachers Study for strong associations between excess mortality and traffic-related exposures.^6,56^ In the previous elemental analyses within the ELAPSE pooled cohort, we observed significantly positive associations for mortality with Cu and Fe in single-pollutant models.^13^ The associations attenuated substantially in models with further adjustment for NO_2_, likely reflecting the common traffic source of these pollutants. Similar to this study, positive associations with mortality were reported in past studies analyzing individual tracers of traffic emissions, which became less stable after adjusting for other traffic-related components such as NO_2_ or organic carbon.^7,8,14^

A majority of the traffic-related PM_2.5_ was explained by BC (25.2%) in our study (Figure 1). There is mounting evidence on associations between long-term exposure to BC and adverse health outcomes.^51,57,58^ In the ELAPSE pooled cohort, we previously found significantly positive associations for BC exposure with mortality and incidence of stroke, asthma, and COPD.^25,59−61^ Health effects of BC were, however, difficult to disentangle from NO_2_ because of their high correlation in concentrations, reflecting conditions in developed countries, where an important source of both pollutants is diesel-powered vehicles. The source apportionment analysis conducted in the present study allowed us to consider the multiple air pollutants from the same source as a group and thus derived more interpretable results.

The oil combustion source component was significantly positively associated with all assessed mortality endpoints in single-source models. HRs reduced moderately and remained statistically significant (borderline significant for CVD mortality) in multisource models. This is consistent with our previous findings that V was most robustly associated with increased mortality risks.^13^ When considered on a per 1 μg/m^3^ comparable basis, the source-labeled residual oil combustion PM_2.5_ had the highest associations with all assessed mortality endpoints (HRs and 95% CIs 1.269 (1.189, 1.354) in the single-source model and 1.128 (1.047, 1.215) in the multisource model for natural mortality) (Table D6,Supporting Information). This suggests that the natural mortality risk estimate for residual oil PM_2.5_ may be about 5 times higher (2.3 if based on the lower and 7.2 based on the upper 95% CI limit) than that for PM_2.5_ mass in general. Previous findings have been mixed on mortality risks associated with residual oil component and its trace elements,^23,62,63^ likely because of the small exposure contrasts combined with low concentrations of residual oil-related particles. In quite a few study areas, residual oil is a less ubiquitous source than motorized traffic. The large population included in the current analysis likely allowed us to detect the potential associations better.

The soil component was positively associated with all assessed mortality endpoints in single-source models. However, these associations reduced to basically unity in multisource models. Crustal materials are often abundant in coarse particles. Our finding of null association is consistent with most previous studies showing little evidence for an association between long-term coarse PM exposure and adverse health effects.^57,64^ The 2019 Integrated Science Assessment (ISA) rated the association between PM_coarse_ exposure and natural-cause mortality as “suggestive”.^65^ A recent analysis conducted in the Medicare enrollees reported significantly positive, but much smaller, association with all-cause mortality for the soil component than for combustion-related components.^66^ The ACS CPS-II found no association with mortality for soil and its elemental tracers (calcium and Si).^56^ Si is a specific tracer for crustal material that is a major component of soil and resuspended road dust. However, a distinction between soil and road dust is often difficult because of the overlapping source profiles.^15^ The California Teachers Study reported adverse cardiovascular associations with long-term exposure to Si, yet the authors interpreted the Si exposure as a proxy either for toxic constituents found in road dust or for exposures to traffic-related pollutants.^7^ The relatively low concentrations of soil component observed in our study suggest that the traffic-associated soil mass from road dust may have been picked up by the traffic component, especially given the large percentage of elemental Si and Fe in the traffic PM_2.5_ profile in Figure 1. Nevertheless, the good practice statements recently provided by the WHO about particles originating from sand and dust storms were to continue monitoring programs and source apportionment activities, health effect analyses, and reduction of exposure.^67^

For the biomass and agriculture component, we observed significantly positive associations with mortality from natural causes, CVDs and LC, and non-significantly negative association with respiratory mortality in single-source models. In multisource models, HRs decreased but remained significantly positive for mortality from natural causes and CVDs, and remained stable for LC mortality. Different from other identified source components, HRs for biomass and agriculture increased from the crude model (model 1) to the model with further adjustment for individual level potential confounders (model 2) (Table D7,Supporting Information). The increase in HR is attributed to the negative correlations between the biomass and agriculture source component and the individual level covariates, indicating population with higher SES or healthier lifestyle tend to be exposed more to PM_2.5_ from biomass and agriculture. We identified the biomass and agriculture component because of its high loading on K (Table 1). However, K is not a unique indicator of wood combustion but can also derive from meat cooking, refuse incineration, and agriculture waste combustion.^16,42^ The California Teachers Study reported positive association between K and IHD mortality,^7^ whereas null association with mortality for K and biomass combustion source category was found in the ACS CPS-II and the Medicare cohort.^56,62^

The industry PM_2.5_ component was positively associated with all assessed mortality outcomes in single source models, but HRs were reduced to unity in multisource models. The observed null associations may be related to the low exposure level and small exposure contrasts exploited in our analyses. Consistently, no association between steel industry-related PM_2.5_ and mortality was found in the Medicare cohort.^62^ The ACS CPS-II reported positive but weak associations with IHD mortality for metal industrial combustion PM_2.5_ (tracers Pb and Zn).^23^

Overall, the present source-specific analysis and the previous individual elemental analysis revealed that particles from residual oil burning (tracers Ni and V) and traffic-related emissions (tracers NO_2_, BC, Cu, and Fe) were most consistently associated with mortality,^13^ agreeing with an analysis in the Netherlands based on dispersion model calculated particle source contributions.^68^

Cumulatively, we found significantly positive associations by air pollution with mortality from natural causes, CVDs and LC. Association with non-malignant respiratory mortality was positive though non-significant. The strongest cumulative risk estimate was for LC mortality (HR: 1.23; 95% CI: 1.10 and 1.38). The cumulative risk estimates were larger than any of the individual source-specific PM_2.5_ HRs resulting from single-source models, supporting that particles from multiple sources were associated with mortality. The cumulative risk was smaller than the sum of HRs, likely attributable to the correlations between PM_2.5_ components.

Sensitivity analyses confirmed the robustness of our findings. Model 1 HRs were almost identical for model 1 and model 3 populations, indicating little selection bias was introduced (Table D8,Supporting Information). When restricting analyses to the follow-up period starting from year 2000 (69% of total person-years at risk, 84% of total deaths), year 2005 (46% of total person-years at risk, 64% of total deaths), year 2008 (32% of total person-years at risk, 47% of total deaths), and year 2010 (23% of total person-years at risk, 33% of total deaths), we observed statistically significant associations between natural mortality and source-specific PM_2.5_, except for PM_2.5_ from biomass and agriculture emissions, which attenuated to unity (Table D9,Supporting Information). When restricting to follow-up starting in 2010, generally smaller HRs with wider CIs were found, likely related to the large reduction in follow-up time and the associated number of events. Applying APCA results derived from the fivefold robustness evaluation analysis resulted in similar source-specific PM_2.5_ associations with natural mortality (Table D10,Supporting Information).

### 3.4 Strengths and Limitations

One strength is the unique and standardized measurement data for PM_2.5_ elemental composition collected from 19 study areas across Europe used for source apportionment analysis. The number of studies on PM component-specific health effects has been small, partly because of the scarcity of measurements relative to regulated pollutants such as PM_2.5_ and NO_2_. Another strength is the large population included in this study, with detailed information on individual and area-level covariates. The pooling of 14 subcohorts and the harmonization of variables across cohorts allowed enhanced statistical power to detect source-specific associations with mortality, especially for source components that have small exposure contrasts. The source apportionment analysis allowed us to assess health effects of PM components in the group context and provided more interpretable results that are more readily translatable into generalizable air quality policy. The correct interpretation of results relies heavily on the reliability of the source apportionment approach. Although the APCA method applied does not quantitatively assess the uncertainties in the mass apportionments, our sensitivity analyses showed robustness of our main APCA approach. Furthermore, an intercomparison among several source apportionment methods has shown that the APCA source apportionment results are consistent across the various methods.^69^ Moreover, an assessment of the contribution by source apportionment to the variability in health effects is a relatively small portion (about 15%) of the uncertainty in the resulting epidemiological health effects estimates.^70^

While it is clear from our findings that source-specific PM_2.5_ differ in their associations with mortality, additional source-specific tracers are needed to be more definitive. We were able to include only nine pollutants in the PCA, whereas source apportionment analyses usually include more tracers. We cannot rule out health risks associated with sources that were not identified by our source apportionment analysis. For example, we could not identify coal burning source, likely because there was not arsenic (As) or selenium (Se) data available to consider in the PCA. Coal combustion PM_2.5_ was found to be most strongly and robustly associated with IHD mortality in the ACS CPS-II and the association between IHD mortality was about five times higher than that for generic PM_2.5_ mass.^23^ Also, more (likely organic) tracers such as levoglucosan need to be analyzed in future work to better separate out the biomass contribution.^71^ We were not able to incorporate organic carbon and heavy metals such as Pb, Cd, and Hg in the analysis, which were reported to be associated with increased mortality risks in a few studies.^7,24^

We had limited ability to investigate the spatial and temporal variability of source components. The air pollution data were collected from 397 monitoring sites across Europe and were clustered (20 or 40 sites per study area). Source of trace elements and their compositions may vary spatially. However, a sensitivity analysis by study area would be unstable and uninformative for our study because of the small number of monitoring sites within each area. Nevertheless, previous source apportionment analyses suggested that the sources of trace elements were relatively stable across European cities.^15^

Another limitation is that the exposure assessment was based on air pollution data in 2010, whereas most included cohorts started their follow-up from mid-1990. However, our sensitivity analyses by restricting recruitment to start years of 2000, 2005, 2008, and 2010 showed robust results, supporting our original analyses in the full cohort. We furthermore note that, for major air pollutants including NO_2_, spatial stability of exposure contrasts over a decade or more has been demonstrated in Europe.^72−74^ While the same data do not exist for source-specific PM, we suspect this holds as well because the spatial distributions of the identified sources have not changed substantially in the already highly developed European urban areas we consider.

## Supporting Information Available

Further elaboration on the APCA; documentation of the process to derive the optimal APCA solution; sensitivity analyses of APCA; factor loadings; percentage of negative source contributions; Spearman correlations between source-specific PM2.5 concentrations; estimated fractional elemental source profiles; descriptives of air pollution measurement data; exposure distribution of source-specific and generic PM2.5; associations of source-specific PM2.5 with mortality; HRs for mortality; associations of source-specific PM2.5 exposures and natural mortality with a restricted follow-up period; and associations between source-specific PM2.5 and natural-cause mortality (PDF)
