# Supplementary material for: Long-Term Exposure to Source-Specific Fine Particles and Mortality—A Pooled Analysis of 14 European Cohorts within the ELAPSE Project
Source: Environ Sci Technol. 2022 Jun 23;56(13):9277–90. doi: 10.1021/acs.est.2c01912 (PMC9261290; doi:10.1021/acs.est.2c01912)
Supplement: Supplementary file 1 — es2c01912_si_001.pdf [file es2c01912_si_001.pdf]

Supporting Information

Long-term exposure to source-specific fine particles and mortality – a pooled analysis of 14 European cohorts within the ELAPSE project

Jie Chen [a], Gerard Hoek [a], Kees de Hoogh [b, c], Sophia Rodopoulou [d], Zorana J. Andersen [e], Tom Bellander [f, g], Jørgen Brandt [h, i], Daniela Fecht [j], Francesco Forastiere [k, l], John Gulliver [m, j], Ole Hertel [n], Barbara Hoffmann [o], Ulla Arthur Hvidtfeldt [p], W.M.Monique Verschuren [q, r], Karl-Heinz Jöckel [s], Jeanette T Jørgensen [e], Klea Katsouyanni [d, l], Matthias Ketzel [h, t], Diego Yacamán Méndez [u, v], Karin Leander [f], Shuo Liu [e], Petter Ljungman [f, w], Elodie Faure [x], Patrik K.E. Magnusson [y], Gabriele Nagel [z], Göran Pershagen [f, g], Annette Peters [aa, ab], Ole Raaschou-Nielsen [p, h], Debora Rizzuto [ac], Evangelia Samoli [d], Yvonne T. van der Schouw [r], Sara Schramm [s], Gianluca Severi [x, ad], Massimo Stafoggia [k, f], Maciej Strak [a, q], Mette Sørensen [p, ae], Anne Tjønneland [p, e], Gudrun Weinmayr [z], Kathrin Wolf [aa], Emanuel Zitt [ag, ah], Bert Brunekreef [a]*, George D. Thurston [ai]*

[a] Institute for Risk Assessment Sciences (IRAS), Utrecht University, 3584 CM Utrecht, the Netherlands
[b] Swiss Tropical and Public Health Institute, 4051 Basel, Switzerland
[c] University of Basel, 4001 Basel, Switzerland
[d] Department of Hygiene, Epidemiology and Medical Statistics, Medical School, National and Kapodistrian University of Athens, 115 27 Athens, Greece
[e] Section of Environment and Health, Department of Public Health, University of Copenhagen, 1165 Copenhagen, Denmark
[f] Institute of Environmental Medicine, Karolinska Institutet, SE-171 77 Stockholm, Sweden
[g] Centre for Occupational and Environmental Medicine, Region Stockholm, 113 65 Stockholm, Sweden
[h] Department of Environmental Science, Aarhus University, Frederiksborgvej 399, DK-4000 Roskilde, Denmark
[i] iClimate – interdisciplinary Center for Climate Change, Aarhus University, Frederiksborgvej 399, DK-4000 Roskilde, Denmark
[j] MRC Centre for Environment and Health, School of Public Health, Imperial College London, Norfolk Place, London, W2 1PG, UK
[k] Department of Epidemiology, Lazio Region Health Service / ASL Roma 1, 00147 Rome, Italy
[l] Environmental Research Group, School of Public Health, Imperial College London, London W2 1PG, UK
[m] Centre for Environmental Health and Sustainability & School of Geography, Geology and the Environment, University of Leicester, LE1 7RH Leicester, UK
[n] Department of Ecoscience, Aarhus University, 4000 Roskilde, Denmark
[o] Institute for Occupational, Social and Environmental Medicine, Centre for Health and Society, Medical Faculty, Heinrich Heine University Düsseldorf, 40001 Düsseldorf, Germany
[p] Danish Cancer Society Research Center, 2100 Copenhagen, Denmark
[q] National Institute for Public Health and the Environment, 3720 BA Bilthoven, the Netherlands
[r] Julius Center for Health Sciences and Primary Care, University Medical Center Utrecht, Utrecht University, 3584 CG Utrecht, the Netherlands.

[s] Institute for Medical Informatics, Biometry and Epidemiology, Medical Faculty, University of Duisburg-Essen, 45259 Essen, Germany
[t] Global Centre for Clean Air Research (GCARE), University of Surrey, GU2 7XH Guildford, United Kingdom
[u] Department of Global Public Health, Karolinska Institutet, 171 77 Stockholm, Sweden
[v] Centre for Epidemiology and Community Medicine, Region Stockholm, 113 65 Stockholm, Sweden
[w] Department of Cardiology, Danderyd University Hospital, 182 88 Stockholm, Sweden
[x] University Paris-Saclay, UVSQ, Inserm, Gustave Roussy, "Exposome and Heredity" team, CESP UMR1018, 94805, Villejuif, France
[y] Department of Medical Epidemiology and Biostatistics, Karolinska Institutet, 171 77 Stockholm, Sweden
[z] Institute of Epidemiology and Medical Biometry, Ulm University, Helmholtzstrasse 22, 89081 Ulm, Germany
[aa] Institute of Epidemiology, Helmholtz Zentrum München, 85764 Neuherberg, Germany
[ab] Chair of Epidemiology, Ludwig Maximilians Universität München, 81377 Munich, Germany
[ac] Department of Neurobiology, Care Sciences, and Society, Karolinska Institutet and Stockholm University, 171 77 Stockholm, Sweden
[ad] Department of Statistics, Computer Science and Applications "G. Parenti" (DISIA), University of Florence, 50121 Firenze FI, Italy
[ae] Department of Natural Science and Environment, Roskilde University, 4000 Roskilde, Denmark
[ag] Agency for Preventive and Social Medicine (aks), 6900 Bregenz, Austria
[ah] Department of Internal Medicine 3, LKH Feldkirch, 6800 Feldkirch, Austria
[ai] Departments of Environmental Medicine and Population Health, New York University Grossman School of Medicine, New York, NY 10010-2598, USA

*These authors contributed equally to this work
Corresponding author: j.chen1@uu.nl

Number of pages: 22
Number of tables: 19
Number of figures: 3

Section A: Further elaboration on the absolute principal component analysis (APCA)

The principal component analysis and subsequent source apportionment work proceeded in two overall stages. We first applied principal component analysis to the entire elemental database (N = 397 observations) to identify the source-related groupings (components) of $PM_{2.5}$ elements. Although conventional factor analysis and principal component analysis are useful for identifying underlying source components based on correlations between elements, they do not directly provide a quantitative apportionment of the $PM_{2.5}$ mass. Therefore, we then apportioned the mass of $PM_{2.5}$ to each of the components, which were then further analyzed in the form of $PM_{2.5}$ contributions in source categories by applying absolute principal component analysis (APCA).[1] In this approach, the individual output values from the principal component analysis (i.e., the principal component scores) were adjusted to provide a quantitative solution relative to a zero pollution concentration case that can be used to apportion mass to each component. Briefly, this approach uses the principal component scores for a zero pollution concentration observation "year" to calculate a baseline value for each component's scores, which are then subtracted from the observed principal component scores to derive "absolute" estimates of each source-specific component's scores. These provided positive indices of attributable component-specific impacts, upon which $PM_{2.5}$ mass concentrations were regressed to allow the attribution of $PM_{2.5}$ mass to each of the principal component analysis derived source-related components. As per Thurston et al.,[2] the $PM_{2.5}$ mass was also ascribed to separate secondary aerosol components in the mass regression step, allowing a fuller and clearer accounting of the $PM_{2.5}$ mass by source category. In this procedure, the mass associated with the secondary aerosols (i.e., sulfates) was therefore apportioned to the principal component analysis identified source components, as possible, with some resulting unexplained secondary sulfate "residual" mass concentrations (e.g., due to long-distance transport), not ascribable to a specific source. It should be noted that, while this APCA method has the advantage that it does not potentially bias the mean $PM_{2.5}$ mass impact estimates by externally constraining all source-specific concentration estimates to be non-negative, it did allow a small percentage of negative contribution estimates for observations where a source impact was at or near zero $\mu g/m^3$. As a result, in this analysis, 9.8% negative source contributions were estimated. During a US EPA Workshop on source apportionment, the APCA approach was found to produce mean apportionment results comparable to other multivariate $PM_{2.5}$ mass source-apportionment approaches [3].

Section B: Documentation of the process to derive the optimal APCA solution

We first performed a varimax rotated principal component analysis (PCA) with the criteria to keep principal components (PCs) have eigenvalues greater than 1.0 after rotation and the cumulative percentage of variance explained larger than 80%, as recommended by Hopke [4]. Varimax rotated PCs have been found to often be more representative of individual underlying sources of variation, compared to unrotated PCs, and are independent of each other [1]. Five PCs were kept and the factor loadings were shown in Table B1. Based on the key tracers in each PC, we identified $PM_{2.5}$ from traffic, residual oil combustion, soil, biomass & agriculture, and industrial sources.

Table B1. Factor loadings for the 5-factor Varimax rotated Principal Component Analysis solution [*S and $PM_{2.5}$ not included in factor analysis]. K_w is soil-adjusted K.

	Traffic	Oil	Soil	Biomass & Agriculture	Industry
NO_2	**0.95**	0.17	-0.01	-0.10	0.09
BC	**0.90**	0.08	0.08	0.25	0.21
Cu	**0.89**	0.07	0.28	0.09	0.15
Fe	**0.78**	0.14	0.51	-0.03	0.18
K_w	0.08	-0.10	0.01	**0.98**	0.11
Ni	0.18	**0.93**	0.19	-0.06	0.10
Si	0.24	0.32	**0.90**	0.02	0.06
V	0.07	**0.95**	0.15	-0.07	0.08
Zn	0.30	0.15	0.08	0.13	**0.93**
*S	0.12	0.47	0.43	0.27	0.26
*$PM_{2.5}$	0.53	0.07	0.22	0.49	0.34
Eigenvalue	3.30	1.96	1.21	1.07	1.00
Cumulative Var	36.7%	58.5%	72.0%	83.9%	95.0%

However, apportioning concentration of $PM_{2.5}$ mass to the five identified source components resulted in 28.7% negative contributions estimated for the soil source component, indicating a non-optimal rotation of the PCs. This is likely due to the forcing of orthogonality (i.e., varimax rotation) when sources are actually correlated with each other in real world. We therefore instead applied Promax rotation, which is an oblique rotation option, that allows control of the extent of intercorrelation of the PCs by specifying the Kappa values – higher values of Kappa lead to higher correlations among PCs with Kappa = 1.0 equals varimax rotation (i.e., orthogonal). We performed additional APCA using Promax rotation specifying varying Kappa values from 1.0 to 4.0 by 0.1 interval. We chose Promax rotated PCA with Kappa = 1.6 as the optimal solution because the negative source contributions were below 10%, while the inter-correlations of the PCs remained below 0.5 (Tables B2 and D2).

We note the 5-factor Promax rotated PCA solution with Kappa = 1.6 resulted in the Eigenvalue of the fifth PC (i.e., industry source component) to be 0.88 after Promax rotation, which is below the pre-specified criterion of 1.0 (Table 1). We therefore tested four-factor PCA solutions with both Varimax and Promax rotations. The four-factor PCA solutions did not result in more identifiable source components and the percentage of negative contributions remained high with increasing Kappa values specified for Promax rotation (Tables B3 to B5). We therefore retained the 5-factor Promax rotated PCA solution with Kappa = 1.6 as the optimal solution.

Table B2 The percentage of negative source contributions when specifying varying Kappa values using 5-factor Promax rotated PCA (%)

**Kappa**	Traffic	Oil	Soil	Biomass & Agriculture	Industry
**1**	1.0	5.5	28.7	1.8	8.1
**1.1**	0.8	4.3	25.7	1.3	4.8
**1.2**	0.5	2.5	20.9	1.3	2.3
**1.3**	0.5	1.5	17.4	1.3	0.8
**1.4**	0.0	1.0	14.1	1.3	0.3
**1.5**	0.0	0.5	11.6	1.3	0.0
**1.6**	0.0	0.5	8.1	1.3	0.0
**1.7**	0.0	0.5	7.1	1.3	0.0
**1.8**	0.0	0.5	5.3	1.3	0.0
**1.9**	0.0	0.5	4.5	1.3	0.0
**2**	0.0	0.3	4.3	1.3	0.0
**2.1**	0.0	0.3	3.0	1.3	0.0
**2.2**	0.0	0.3	2.5	1.3	0.0
**2.3**	0.0	0.3	2.3	1.3	0.0
**2.4**	0.0	0.3	1.5	1.3	0.0
**2.5**	0.0	0.0	1.5	1.3	0.0
**2.6**	0.0	0.0	1.5	1.3	0.0
**2.7**	0.0	0.0	1.5	1.3	0.0
**2.8**	0.0	0.0	1.5	1.3	0.0
**2.9**	0.0	0.0	1.3	1.3	0.0
**3**	0.0	0.0	1.3	1.3	0.0
**3.1**	0.0	0.0	1.3	1.3	0.0
**3.2**	0.0	0.0	1.3	1.3	0.0
**3.3**	0.0	0.0	1.3	1.3	0.0
**3.4**	0.0	0.0	1.3	1.3	0.0
**3.5**	0.0	0.0	1.0	1.3	0.0
**3.6**	0.0	0.0	1.0	1.3	0.0
**3.7**	0.0	0.0	1.0	1.3	0.0
**3.8**	0.0	0.0	0.8	1.3	0.0
**3.9**	0.0	0.0	0.8	1.3	0.0
**4**	0.0	0.0	0.8	1.3	0.0

Table B3. Factor loadings for the 4-factor Varimax rotated Principal Component Analysis solution [*S and $PM_{2.5}$ not included in factor analysis]. K_w is soil-adjusted K.

	Traffic	Oil	Biomass & Agriculture	Soil
NO_2	**0.94**	0.14	-0.11	0.00
BC	**0.90**	0.08	0.27	0.06
Cu	**0.91**	0.08	0.11	0.25
Fe	**0.83**	0.20	0.03	0.42
K_w	0.05	-0.16	**0.94**	0.08
Ni	0.18	**0.91**	-0.06	0.20
Si	0.30	0.39	0.07	**0.81**
V	0.07	**0.94**	-0.08	0.15
Zn	0.51	0.42	0.47	-0.27
*S	0.18	0.53	0.35	0.35
*$PM_{2.5}$	0.57	0.12	0.57	0.15
Eigenvalue	3.60	2.13	1.21	1.05
Cumulative Var	40.0%	63.7%	77.2%	88.8%

Table B4. The percentage of negative source contributions when specifying varying Kappa values using 4-factor Promax rotated PCA (%)

**Kappa**	Traffic	Oil	Biomass & Agriculture	Soil
**1**	2.3	8.6	0.8	30.5
**1.1**	1.8	6.8	0.8	27.7
**1.2**	1.3	6.0	0.5	26.2
**1.3**	0.5	4.0	0.5	25.2
**1.4**	0.5	3.3	0.5	24.9
**1.5**	0.0	2.3	0.5	23.9
**1.6**	0.0	1.5	0.3	23.4
**1.7**	0.0	0.8	0.3	22.2
**1.8**	0.0	0.0	0.3	21.2
**1.9**	0.0	0.0	0.3	20.9
**2**	0.0	0.0	0.3	20.9
**2.1**	0.0	0.0	0.3	21.2
**2.2**	0.0	0.0	0.3	21.7
**2.3**	0.0	0.0	0.3	21.7
**2.4**	0.0	0.0	0.3	22.7
**2.5**	0.0	0.0	0.3	23.4
**2.6**	0.0	0.0	0.3	23.7
**2.7**	0.0	0.0	0.3	24.4
**2.8**	0.0	0.0	0.3	24.4
**2.9**	0.0	0.0	0.3	24.9
**3**	0.0	0.0	0.3	25.2
**3.1**	0.0	0.0	0.3	25.4
**3.2**	0.0	0.0	0.3	26.4
**3.3**	0.0	0.0	0.3	27.2
**3.4**	0.0	0.0	0.3	27.7

**3.5**	0.0	0.0	0.3	27.7
**3.6**	0.0	0.0	0.3	28.0
**3.7**	0.0	0.0	0.3	28.2
**3.8**	0.0	0.0	0.3	28.5
**3.9**	0.0	0.0	0.3	29.0
**4**	0.0	0.0	0.3	30.0

Table B5. Factor loadings for the 4-factor Promax rotated Principal Component Analysis solution (Kappa = 1.9) [*S and $PM_{2.5}$ not included in factor analysis]. K_w is soil-adjusted K.

	Traffic	Oil	Biomass & Agriculture	Soil
NO_2	**0.98**	0.01	-0.19	-0.06
BC	**0.90**	-0.03	0.19	0.02
Cu	**0.91**	-0.06	0.04	0.20
Fe	**0.81**	0.06	-0.02	0.38
K_w	-0.05	-0.13	**0.95**	0.11
Ni	0.04	**0.90**	-0.05	0.15
Si	0.18	0.31	0.08	**0.79**
V	-0.08	**0.95**	-0.06	0.11
Zn	0.43	0.40	0.43	-0.31
*S	0.03	0.52	0.36	0.33
*$PM_{2.5}$	0.50	0.07	0.53	0.13
Eigenvalue	3.46	2.00	1.19	0.96
Cumulative Var	38.4%	60.6%	73.8%	84.5%

Section C: Sensitivity analyses of APCA

Table C1. Factor loadings for the 5-factor Promax rotated PCA – five-fold robustness evaluation each used 80% of the monitoring sites

1) Evaluation set 1 (N=325), Kappa = 1.6

	Traffic	Oil	Soil	Biomass & Agriculture	Industry
NO_2	**0.97**	0.09	-0.09	-0.11	0.00
BC	**0.89**	0.04	0.00	0.22	0.08
Cu	**0.87**	-0.03	0.21	0.05	0.05
Fe	**0.71**	0.01	0.44	-0.06	0.12
K_w	0.02	-0.02	0.00	**0.99**	0.02
Ni	0.10	**0.91**	0.11	-0.01	0.03
Si	0.10	0.20	**0.89**	0.01	0.00
V	-0.02	**0.96**	0.05	-0.01	0.03
Zn	0.16	0.07	0.01	0.03	**0.92**
*S	-0.01	0.41	0.38	0.25	0.23
*$PM_{2.5}$	0.46	0.04	0.16	0.45	0.23
Eigenvalue	3.05	1.80	1.05	1.04	0.87
Cumulative Var	33.9%	53.9%	65.6%	77.1%	86.8%

2) Evaluation set 2 (N=307), Kappa = 1.5

	Traffic	Oil	Soil	Biomass & Agriculture	Industry
NO_2	**0.97**	0.10	-0.07	-0.11	0.00
BC	**0.87**	0.03	0.02	0.23	0.09
Cu	**0.87**	-0.02	0.20	0.07	0.07
Fe	**0.69**	0.02	0.48	-0.06	0.15
K_w	0.03	-0.02	0.00	**0.98**	0.03
Ni	0.10	**0.91**	0.11	-0.01	0.04
Si	0.10	0.22	**0.89**	0.02	-0.01
V	-0.02	**0.96**	0.07	-0.01	0.02
Zn	0.18	0.06	0.01	0.05	**0.92**
*S	0.02	0.44	0.41	0.26	0.18
*$PM_{2.5}$	0.47	0.04	0.18	0.45	0.22
Eigenvalue	2.99	1.81	1.08	1.05	0.89
Cumulative Var	33.2%	53.3%	65.3%	76.9%	86.7%

3) Evaluation set 3 (N=318), Kappa = 1.7

	Traffic	Oil	Soil	Biomass & Agriculture	Industry
NO_2	**0.97**	0.13	-0.11	-0.11	-0.02
BC	**0.86**	0.03	0.02	0.23	0.09
Cu	**0.82**	-0.01	0.26	0.05	0.05
Fe	**0.67**	0.01	0.48	-0.08	0.10
K_w	0.02	-0.04	-0.01	**0.98**	0.02
Ni	0.09	**0.90**	0.11	-0.01	0.02
Si	0.08	0.21	**0.87**	0.02	-0.01
V	0.02	**0.93**	0.07	-0.02	0.04
Zn	0.12	0.07	0.01	0.03	**0.93**
*S	-0.03	0.39	0.40	0.24	0.22
*$PM_{2.5}$	0.38	0.03	0.20	0.45	0.27
Eigenvalue	2.85	1.75	1.09	1.04	0.89
Cumulative Var	31.6%	51.0%	63.2%	74.8%	84.6%

4) Evaluation set 4 (N=316), Kappa = 1.7

	Traffic	Oil	Soil	Biomass & Agriculture	Industry
NO_2	**0.98**	0.09	-0.10	-0.11	0.01
BC	**0.87**	0.02	-0.01	0.21	0.12
Cu	**0.87**	-0.03	0.20	0.06	0.03
Fe	**0.77**	0.03	0.42	-0.06	0.03
K_w	0.02	-0.01	0.00	**0.99**	0.01
Ni	0.09	**0.92**	0.08	0.00	0.02
Si	0.12	0.18	**0.88**	0.01	0.01
V	-0.02	**0.96**	0.05	-0.01	0.02
Zn	0.14	0.05	0.01	0.01	**0.93**
*S	-0.03	0.44	0.36	0.31	0.20
*$PM_{2.5}$	0.43	0.04	0.14	0.49	0.25
Eigenvalue	3.12	1.80	1.05	1.01	0.89
Cumulative Var	34.7%	54.7%	66.3%	77.6%	87.5%

5) Evaluation set 5 (N=322), Kappa = 1.6

	Traffic	Oil	Soil	Biomass & Agriculture	Industry
NO_2	**0.97**	0.10	-0.09	-0.13	0.01
BC	**0.87**	0.03	-0.01	0.23	0.11
Cu	**0.87**	-0.03	0.22	0.08	0.02
Fe	**0.71**	0.01	0.46	-0.05	0.09
K_w	0.02	-0.03	-0.01	**0.98**	0.04
Ni	0.10	**0.92**	0.09	-0.01	0.02
Si	0.09	0.18	**0.90**	0.01	0.00
V	-0.03	**0.96**	0.05	-0.01	0.01
Zn	0.15	0.04	0.01	0.05	**0.93**
*S	-0.01	0.45	0.37	0.30	0.18
*$PM_{2.5}$	0.44	0.03	0.15	0.45	0.28
Eigenvalue	3.01	1.81	1.10	1.05	0.89
Cumulative Var	33.5%	53.6%	65.8%	77.4%	87.3%

*S and $PM_{2.5}$ not included in factor analysis. K_w is soil-adjusted K.

Table C2. Spearman correlations between source-specific $PM_{2.5}$ concentrations at monitoring sites – five-fold robustness evaluation each used 80% of monitoring sites

	Traffic	Oil	Soil	Biomass & Agriculture	Industry
Evaluation set 1 (N = 325)					
Traffic	1	0.23	0.19	0.10	0.42
Oil		1	0.32	-0.16	0.06
Soil			1	0.17	0.22
Biomass & Agriculture				1	0.39
Evaluation set 2 (N = 307)					
Traffic	1	0.24	0.17	0.06	0.37
Oil		1	0.29	-0.14	0.05
Soil			1	0.21	0.20
Biomass & Agriculture				1	0.35
Evaluation set 3 (N = 318)					
Traffic	1	0.26	0.23	0.14	0.41
Oil		1	0.29	-0.22	0.06
Soil			1	0.21	0.30
Biomass & Agriculture				1	0.45
Evaluation set 4 (N = 316)					
Traffic	1	0.25	0.14	0.24	0.48
Oil		1	-0.14	0.33	0.12
Soil			1	0.19	0.40
Biomass & Agriculture				1	0.25
Evaluation set 5 (N = 322)					
Traffic	1	0.20	0.21	0.10	0.42
Oil		1	0.31	-0.21	0.06
Soil			1	0.16	0.24
Biomass & Agriculture				1	0.37

Table C3. Factor loadings for the 5-factor Promax rotated Principal Component Analysis solution including S (Kappa = 1.2) [*$PM_{2.5}$ not included in factor analysis]. K_w is soil-adjusted K.

	Traffic	Oil	Soil	Biomass & Agriculture	Industry
NO_2	**0.95**	0.15	-0.06	-0.08	0.05
BC	**0.87**	0.06	0.05	0.26	0.16
Cu	**0.88**	0.02	0.24	0.08	0.09
Fe	**0.78**	0.07	0.46	-0.06	0.12
K_w	0.07	-0.08	0.01	**0.96**	0.05
Ni	0.16	**0.90**	0.16	-0.06	0.05
S	0.01	0.44	0.59	0.32	0.27
Si	0.25	0.21	**0.86**	-0.05	-0.01
V	0.04	**0.93**	0.14	-0.05	0.05
Zn	0.26	0.09	0.07	0.06	**0.90**
*$PM_{2.5}$	0.47	0.05	0.27	0.50	0.30
Eigenvalue	3.21	1.96	1.41	1.13	0.94
Cumulative Var	32.1%	51.7%	65.8%	77.0%	86.5%

Table C4. Factor loadings for the 5-factor Promax rotated Principal Component Analysis solution without adjusting K for soil-associated K (Kappa = 1.9) [*S and $PM_{2.5}$ not included in factor analysis].

	Traffic	Oil	Soil	Biomass & Agriculture	Industry
NO_2	**0.99**	0.10	-0.07	-0.11	-0.03
BC	**0.88**	0.02	-0.08	0.25	0.07
Cu	**0.86**	-0.05	0.20	0.06	0.03
Fe	**0.69**	-0.01	0.49	-0.06	0.08
K	0.03	0.00	0.06	**0.97**	0.02
Ni	0.08	**0.91**	0.09	0.00	0.02
Si	0.05	0.18	**0.87**	0.09	-0.02
V	-0.03	**0.97**	0.03	0.01	0.01
Zn	0.11	0.04	0.00	0.02	**0.95**
*S	-0.05	0.41	0.30	0.31	0.20
*$PM_{2.5}$	0.42	0.01	0.03	0.48	0.24
Eigenvalue	2.99	1.81	1.07	1.03	0.91
Cumulative Var	33.2%	53.4%	65.2%	76.6%	86.7%

Evaluation set 1

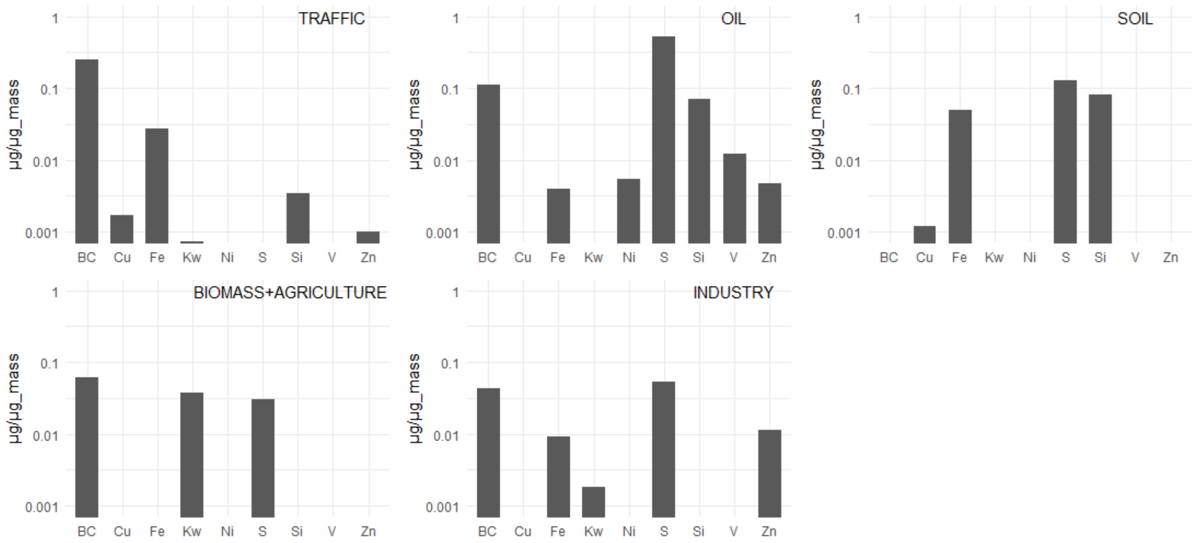

Evaluation set 2

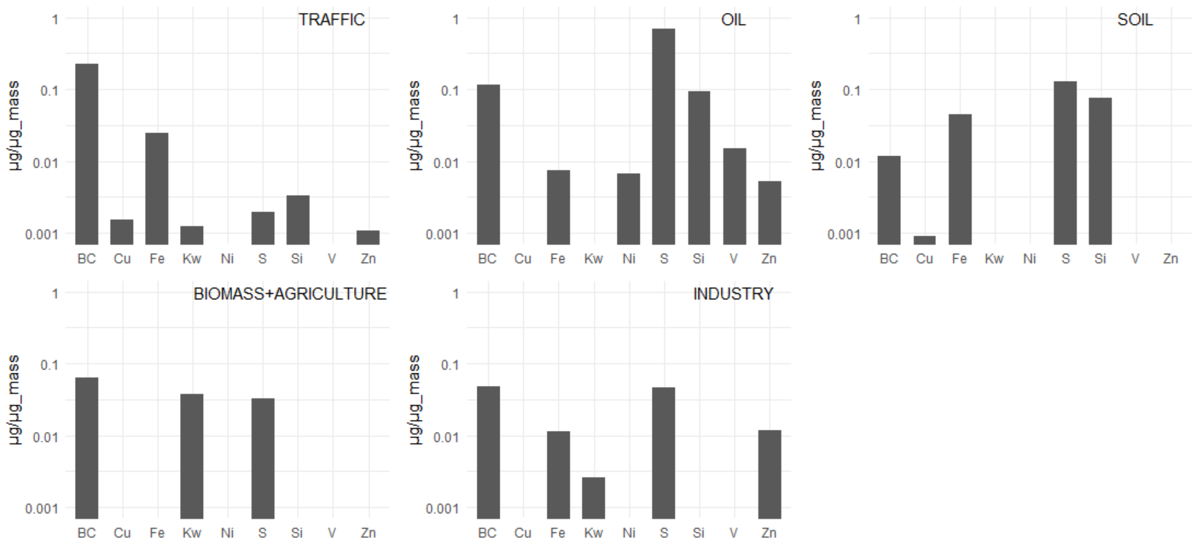

Evaluation set 3

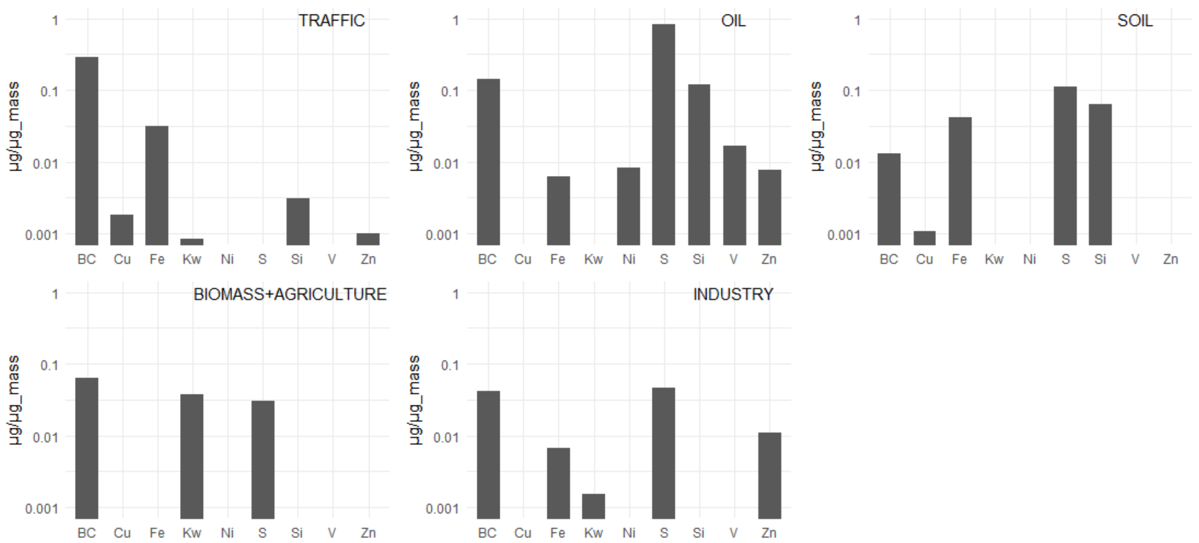

Evaluation set 4

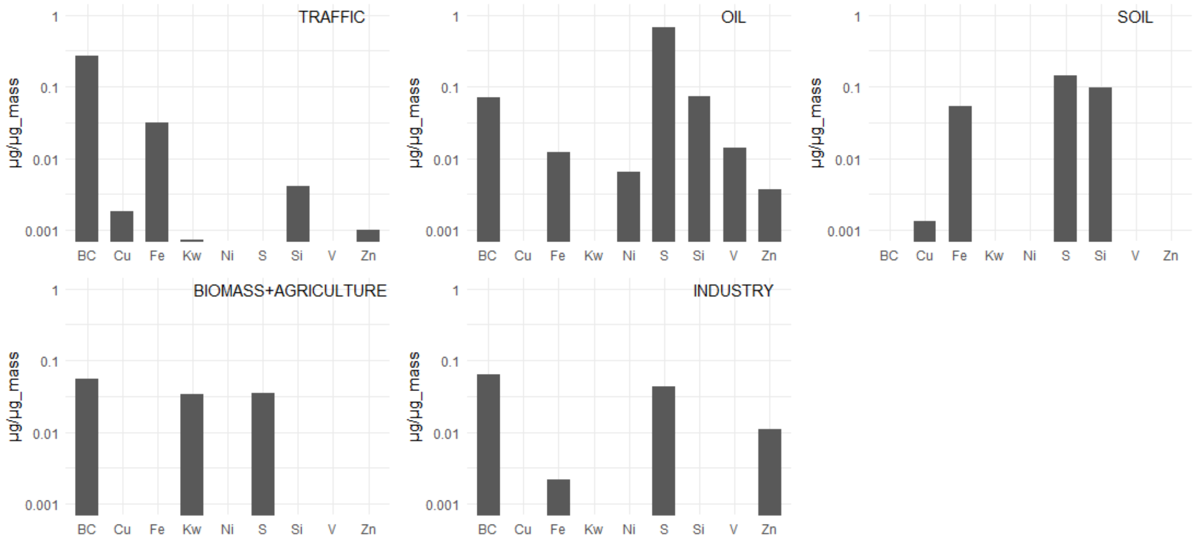

Evaluation set 5

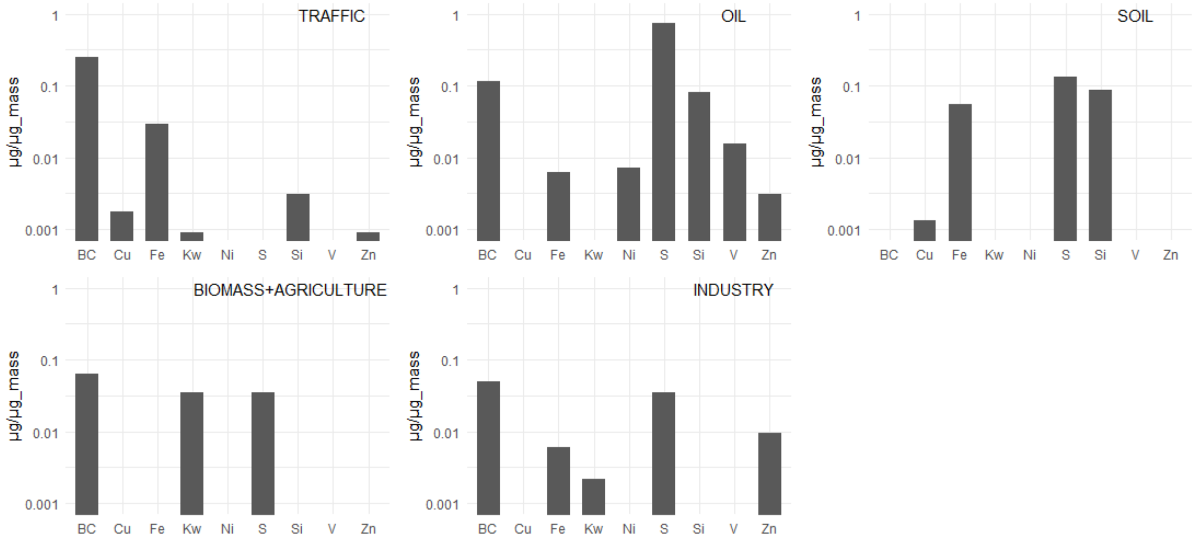

Figure C1. Estimated fractional elemental source profiles of identified source-specific $PM_{2.5}$ – five-fold robustness evaluation each used 80% of monitoring sites

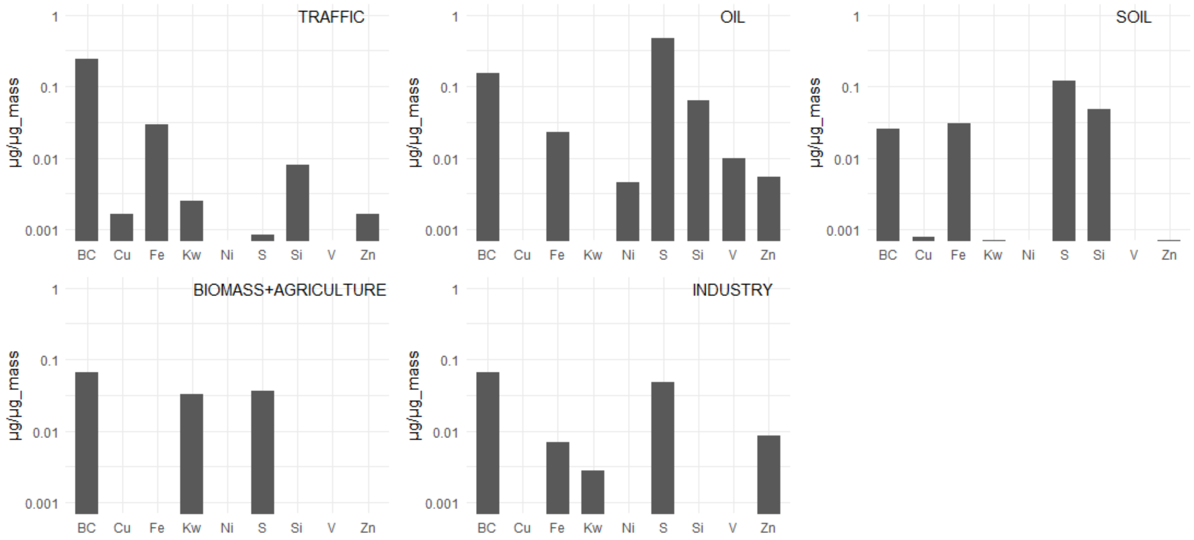

Figure C2. Estimated fractional elemental source profiles of identified source-specific $PM_{2.5}$ – sensitivity analysis with S included in PCA

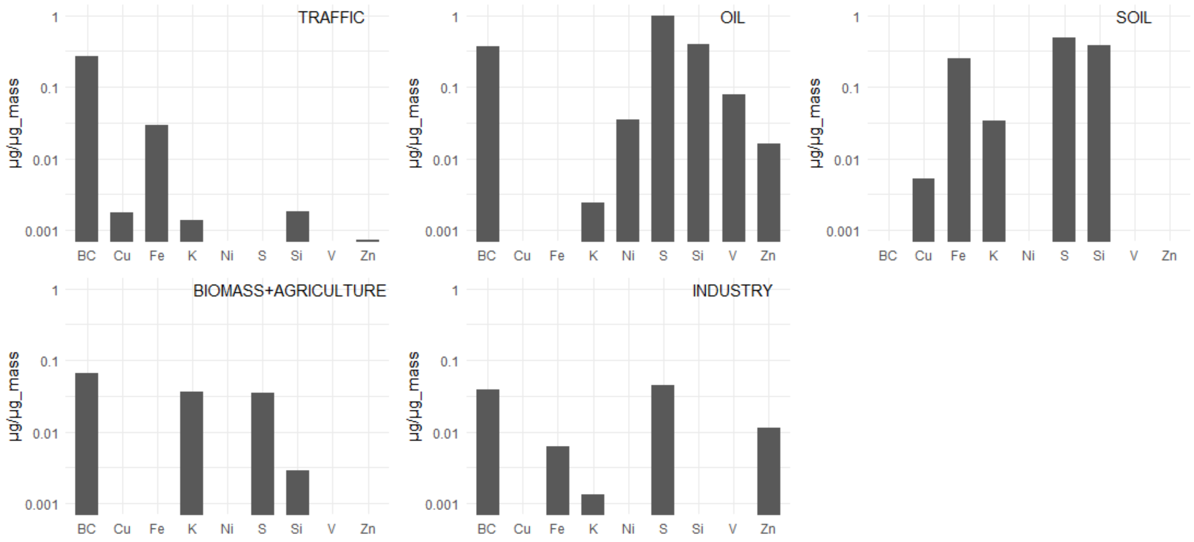

Figure C3. Estimated fractional elemental source profiles of identified source-specific $PM_{2.5}$ – sensitivity analysis without adjusting for soil-associated K in PCA

Section D: Supplementary tables and figures

Table D1. Descriptives of air pollution measurement data (N = 397)

	ng/m^3								μg/m^3		0.5×10^{-5}/m
	Cu	Fe	Kw	Ni	S	Si	V	Zn	$PM_{2.5}$ mass	NO_2	BC
**Mean**	**6.2**	**142.3**	**138.7**	**1.6**	**919.0**	**137.3**	**2.9**	**26.0**	**15.95**	28.1	1.8
**SD**	**5.0**	**101.6**	**96.6**	**1.5**	**317.3**	**86.0**	**3.1**	**16.8**	**5.75**	16.2	0.8
IQR	5.0	107.1	138.3	1.6	385.3	116.4	2.3	17.9	7.70	19.2	1.0
P5	1.1	35.8	39.4	0.1	502.3	40.4	0.3	10.1	7.95	9.3	0.9
P25	2.8	72.7	67.7	0.6	697.3	70.1	1.0	14.6	11.72	16.3	1.3
Median	4.7	108.9	97.0	1.2	877.1	112.6	1.9	20.8	15.74	24.9	1.7
P75	7.8	179.8	206.0	2.1	1082.6	186.5	3.3	32.4	19.39	35.5	2.3
P95	15.8	327.6	322.7	4.7	1587.8	307.6	9.1	57.6	26.24	59.3	3.4

Table D2. Spearman correlations between source-specific $PM_{2.5}$ concentrations at monitoring sites (N = 397)

	Traffic	Oil	Soil	Biomass & Agriculture	Industry
Traffic	1	0.24	0.20	0.11	0.42
Oil		1	0.30	-0.18	0.07
Soil			1	0.19	0.24
Biomass & Agriculture				1	0.39

Table D3. Exposure distribution of source-specific and the generic $PM_{2.5}$ in the pooled cohort (μg/m^3; N = 323,782)

	Traffic	Oil	Soil	Biomass & Agriculture	Industry	$PM_{2.5}$ mass
**Mean**	4.01	0.20	0.96	3.70	1.78	15.02
**SD**	2.28	0.20	0.86	2.69	1.32	3.22
IQR	2.86	0.25	0.95	4.32	1.09	4.49
P5	0.43	-0.01	-0.22	-0.29	0.35	8.62
P25	2.45	0.06	0.42	1.51	1.14	12.83
Median	4.05	0.12	0.90	3.73	1.60	15.51
P75	5.31	0.31	1.37	5.83	2.23	17.32
P95	7.76	0.60	2.35	7.97	3.33	19.43

Table D4. Spearman correlations between source-specific $PM_{2.5}$ exposures at baseline residential addresses (median of cohort-specific correlations; μg/m^3; N = 323,782)

	Traffic	Oil	Soil	Biomass & Agriculture	Industry
Traffic	1	0.27	0.66	-0.48	0.13
Oil		1	0.37	0.01	0.64
Soil			1	-0.33	0.57
Biomass & Agriculture				1	0.12

Table D5. Associations of source-specific $PM_{2.5}$ with mortality from natural-cause (NAT), cardiovascular diseases (CVD), non-malignant respiratory diseases (RESP) and lung cancer (LC) derived from the fully adjusted model[a]. HRs (95% CI) are presented for per IQR increase in exposures.

	Single-source HR	Multi-source HR
	**Natural mortality (N = 46,640)**	
Traffic	1.08 (1.07, 1.10)	1.06 (1.04, 1.08)
Oil	1.06 (1.04, 1.08)	1.03 (1.01, 1.05)
Soil	1.05 (1.04, 1.06)	1.01 (0.99, 1.03)
Bio&Agr	1.07 (1.04, 1.09)	1.04 (1.01, 1.07)
Industry	1.04 (1.02, 1.05)	1.00 (0.98, 1.02)
$PM_{2.5}$ mass	1.12 (1.10, 1.14)	-
Cumulative Risk	-	1.14 (1.11, 1.18)
	**Cardiovascular mortality (N = 15,492)**	
Traffic	1.09 (1.06, 1.11)	1.06 (1.02, 1.10)
Oil	1.06 (1.03, 1.10)	1.03 (0.98, 1.07)
Soil	1.05 (1.03, 1.07)	1.01 (0.98, 1.05)
Bio&Agr	1.09 (1.04, 1.13)	1.06 (1.00, 1.12)
Industry	1.04 (1.02, 1.06)	0.99 (0.95, 1.02)
$PM_{2.5}$ mass	1.12 (1.09, 1.16)	-
Cumulative Risk	-	1.15 (1.09, 1.21)
	**Non-malignant Respiratory mortality (N = 2,846)**	
Traffic	1.09 (1.02, 1.15)	1.09 (1.01, 1.17)
Oil	1.10 (1.04, 1.16)	1.08 (1.01, 1.16)
Soil	1.03 (0.98, 1.09)	0.98 (0.91, 1.05)
Bio&Agr	0.92 (0.82, 1.02)	0.90 (0.79, 1.03)
Industry	1.01 (0.96, 1.07)	0.99 (0.91, 1.07)
$PM_{2.5}$ mass	1.04 (0.96, 1.14)	-
Cumulative Risk	-	1.03 (0.90, 1.17)
	**Lung cancer mortality (N = 3,776)**	
Traffic	1.07 (1.02, 1.13)	1.03 (0.97, 1.10)
Oil	1.07 (1.02, 1.13)	1.06 (1.00, 1.12)
Soil	1.05 (1.00, 1.09)	1.02 (0.97, 1.08)
Bio&Agr	1.11 (1.01, 1.23)	1.12 (1.00, 1.25)
Industry	1.04 (1.00, 1.08)	0.99 (0.94, 1.04)
$PM_{2.5}$ mass	1.17 (1.08, 1.25)	-
Cumulative Risk	-	1.23 (1.10, 1.38)

Total number of observations= 323,782; person-years at risk =6,317,235.

[a]Model 3 adjusted for age, sub-cohort id, sex, year of enrollment, smoking (status, duration, intensity, $intensity^2$), BMI categories, marital status, employment status and 2001 area-level mean income

HRs (95% CI) are presented for the increment of the interquartile range for each exposure in the pooled cohort: Traffic 2.86 $\mu g/m^3$; Oil 0.25 $\mu g/m^3$; Soil 0.95 $\mu g/m^3$; Biomass & Agriculture 4.32 $\mu g/m^3$; Industry 1.09 $\mu g/m^3$; $PM_{2.5}$ mass 4.49 $\mu g/m^3$ (Table D3)

Table D6. Associations of source-specific $PM_{2.5}$ with mortality from natural-cause (NAT), cardiovascular diseases (CVD), non-malignant respiratory diseases (RESP) and lung cancer (LC). HRs (95% CI) are presented for per 1 µg/m^3 increase in $PM_{2.5}$

	Single-source HR	Multi-source HR
	**Natural mortality (N = 46,640)**	
Traffic	1.03 (1.02, 1.03)	1.02 (1.01, 1.03)
Oil	1.27 (1.19, 1.35)	1.13 (1.05, 1.22)
Soil	1.05 (1.04, 1.06)	1.01 (0.99, 1.03)
Bio&Agr	1.02 (1.01, 1.02)	1.01 (1.00, 1.02)
Industry	1.03 (1.02, 1.04)	1.00 (0.98, 1.01)
$PM_{2.5}$ mass	1.03 (1.02, 1.03)	-
	**Cardiovascular mortality (N = 15,492)**	
Traffic	1.03 (1.02, 1.04)	1.02 (1.01, 1.03)
Oil	1.27 (1.10, 1.45)	1.10 (0.94, 1.29)
Soil	1.05 (1.03, 1.08)	1.01 (0.98, 1.05)
Bio&Agr	1.02 (1.01, 1.03)	1.01 (1.00, 1.03)
Industry	1.04 (1.02, 1.06)	0.99 (0.95, 1.02)
$PM_{2.5}$ mass	1.03 (1.02, 1.03)	-
	**Non-malignant Respiratory mortality (N = 2,846)**	
Traffic	1.03 (1.01, 1.05)	1.03 (1.00, 1.06)
Oil	1.45 (1.15, 1.82)	1.38 (1.06, 1.79)
Soil	1.04 (0.98, 1.09)	0.98 (0.91, 1.05)
Bio&Agr	0.98 (0.96, 1.01)	0.98 (0.95, 1.01)
Industry	1.01 (0.96, 1.06)	0.99 (0.92, 1.06)
$PM_{2.5}$ mass	1.01 (0.99, 1.03)	-
	**Lung cancer mortality (N = 3,776)**	
Traffic	1.02 (1.01, 1.04)	1.01 (0.99, 1.04)
Oil	1.32 (1.08, 1.60)	1.24 (0.99, 1.54)
Soil	1.05 (1.00, 1.10)	1.02 (0.96, 1.09)
Bio&Agr	1.03 (1.00, 1.05)	1.03 (1.00, 1.05)
Industry	1.03 (1.00, 1.07)	0.99 (0.94, 1.04)
$PM_{2.5}$ mass	1.03 (1.02, 1.05)	-

Total number of observations= 323,782; person-years at risk =6,317,235.

The main model adjusted for sub-cohort identification, age, sex, year of enrollment, smoking (status, duration, intensity, and intensity^2), BMI categories, marital status, employment status, and 2001 area-level mean income.

Table D7. Associations of source-specific $PM_{2.5}$ with mortality from natural-cause (NAT), cardiovascular diseases (CVD), non-malignant respiratory diseases (RESP) and lung cancer (LC) with increasing control for covariates

	Model 1 HR	Model 2 HR	Model 3 HR
		**Natural mortality (N = 46,640)**	
Traffic	1.11 (1.09, 1.12)	1.07 (1.05, 1.08)	1.08 (1.07, 1.10)
Oil	1.13 (1.11, 1.15)	1.07 (1.05, 1.08)	1.06 (1.04, 1.08)
Soil	1.09 (1.08, 1.10)	1.05 (1.03, 1.06)	1.05 (1.04, 1.06)
Bio&Agr	1.05 (1.02, 1.07)	1.06 (1.03, 1.08)	1.07 (1.04, 1.09)
Industry	1.05 (1.04, 1.06)	1.03 (1.02, 1.04)	1.04 (1.02, 1.05)
$PM_{2.5}$ mass	1.14 (1.12, 1.16)	1.11 (1.09, 1.13)	1.12 (1.10, 1.14)
		**Cardiovascular mortality (N = 15,492)**	
Traffic	1.09 (1.06, 1.12)	1.06 (1.03, 1.09)	1.09 (1.06, 1.11)
Oil	1.12 (1.08, 1.16)	1.07 (1.03, 1.10)	1.06 (1.03, 1.10)
Soil	1.08 (1.05, 1.10)	1.04 (1.02, 1.07)	1.05 (1.03, 1.07)
Bio&Agr	1.06 (1.02, 1.11)	1.07 (1.02, 1.11)	1.09 (1.04, 1.13)
Industry	1.05 (1.02, 1.07)	1.03 (1.01, 1.05)	1.04 (1.02, 1.06)
$PM_{2.5}$ mass	1.12 (1.09, 1.16)	1.11 (1.08, 1.15)	1.12 (1.09, 1.16)
		**Non-malignant Respiratory mortality (N = 2,846)**	
Traffic	1.11 (1.05, 1.18)	1.04 (0.98, 1.10)	1.09 (1.02, 1.15)
Oil	1.23 (1.16, 1.30)	1.13 (1.07, 1.19)	1.10 (1.04, 1.16)
Soil	1.13 (1.08, 1.19)	1.04 (0.99, 1.09)	1.03 (0.98, 1.09)
Bio&Agr	0.84 (0.75, 0.94)	0.88 (0.79, 0.98)	0.92 (0.82, 1.02)
Industry	1.05 (1.00, 1.10)	1.00 (0.94, 1.05)	1.01 (0.96, 1.07)
$PM_{2.5}$ mass	1.06 (0.97, 1.15)	1.02 (0.94, 1.11)	1.04 (0.96, 1.14)
		**Lung cancer mortality (N = 3,776)**	
Traffic	1.15 (1.09, 1.21)	1.05 (1.00, 1.10)	1.07 (1.02, 1.13)
Oil	1.19 (1.14, 1.25)	1.08 (1.03, 1.14)	1.07 (1.02, 1.13)
Soil	1.16 (1.12, 1.21)	1.05 (1.01, 1.10)	1.05 (1.00, 1.09)
Bio&Agr	1.07 (0.97, 1.17)	1.10 (1.00, 1.21)	1.11 (1.01, 1.23)
Industry	1.08 (1.04, 1.12)	1.03 (0.99, 1.07)	1.04 (1.00, 1.08)
$PM_{2.5}$ mass	1.23 (1.14, 1.32)	1.16 (1.07, 1.24)	1.17 (1.08, 1.25)

Total number of observations= 323,782; person-years at risk =6,317,235.

Model 1 adjusted for age, sub-cohort id, sex and year of enrollment; Model 2 further adjusted for smoking (status, duration, intensity, $intensity^2$), BMI categories, marital status and employment status; Model 3 further adjusted for 2001 area-level mean income

HRs (95% CI) are presented for the increment of the interquartile range for each exposure in the pooled cohort: Traffic 2.86 $\mu g/m^3$; Oil 0.25 $\mu g/m^3$; Soil 0.95 $\mu g/m^3$; Biomass & Agriculture 4.32 $\mu g/m^3$; Industry 1.09 $\mu g/m^3$; $PM_{2.5}$ mass 4.49 $\mu g/m^3$ (Table D3)

Table D8. Hazard ratios (HRs) for mortality from natural-cause, cardiovascular diseases, non-malignant respiratory diseases and lung cancer with source-specific $PM_{2.5}$ derived from Model 1 in the full population and the population included in main analyses

	Model 1 population (N = 378,979)	Model 3 population (N = 323,782)
	**Natural mortality**	
Traffic	1.11 (1.09, 1.12)	1.11 (1.09, 1.12)
Oil	1.13 (1.12, 1.15)	1.13 (1.11, 1.15)
Soil	1.08 (1.07, 1.09)	1.09 (1.08, 1.10)
Bio&Agr	1.05 (1.03, 1.08)	1.05 (1.02, 1.07)
Industry	1.05 (1.04, 1.06)	1.05 (1.04, 1.06)
$PM_{2.5}$ mass	1.13 (1.11, 1.16)	1.14 (1.12, 1.16)
	**Cardiovascular mortality**	
Traffic	1.09 (1.06, 1.11)	1.09 (1.06, 1.12)
Oil	1.13 (1.10, 1.17)	1.12 (1.08, 1.16)
Soil	1.06 (1.04, 1.09)	1.08 (1.05, 1.10)
Bio&Agr	1.07 (1.03, 1.11)	1.06 (1.02, 1.11)
Industry	1.05 (1.03, 1.07)	1.05 (1.02, 1.07)
$PM_{2.5}$ mass	1.12 (1.09, 1.15)	1.12 (1.09, 1.16)
	**Non-malignant Respiratory mortality**	
Traffic	1.13 (1.07, 1.19)	1.11 (1.05, 1.18)
Oil	1.21 (1.15, 1.28)	1.23 (1.16, 1.30)
Soil	1.13 (1.08, 1.18)	1.13 (1.08, 1.19)
Bio&Agr	0.85 (0.76, 0.94)	0.84 (0.75, 0.94)
Industry	1.04 (1.00, 1.09)	1.05 (1.00, 1.10)
$PM_{2.5}$ mass	1.05 (0.97, 1.13)	1.06 (0.97, 1.15)
	**Lung cancer mortality**	
Traffic	1.14 (1.09, 1.19)	1.15 (1.09, 1.21)
Oil	1.19 (1.14, 1.25)	1.19 (1.14, 1.25)
Soil	1.14 (1.10, 1.18)	1.16 (1.12, 1.21)
Bio&Agr	1.07 (0.98, 1.17)	1.07 (0.97, 1.17)
Industry	1.08 (1.04, 1.11)	1.08 (1.04, 1.12)
$PM_{2.5}$ mass	1.19 (1.12, 1.28)	1.23 (1.14, 1.32)

Person-years at risk =7,291,866 in Model 1 population; person-years at risk = 6,317,235 in Model 3 population

Model 1 adjusted for age, sub-cohort id, sex and year of enrollment

HRs (95% CI) are presented for the increment of the interquartile range for each exposure in the pooled cohort: Traffic 2.86 $\mu g/m^3$; Oil 0.25 $\mu g/m^3$; Soil 0.95 $\mu g/m^3$; Biomass & Agriculture 4.32 $\mu g/m^3$; Industry 1.09 $\mu g/m^3$; $PM_{2.5}$ mass 4.49 $\mu g/m^3$ (Table D3)

Table D9. Associations of source-specific $PM_{2.5}$ exposures and natural mortality with restricted follow-up period

Exposure	Hazard Ratio (95% confidence interval) per IQR increase				
	Full follow-up[a]	After 2000[b]	After 2005[c]	After 2008[d]	After 2010[e]
Traffic	1.08 (1.07, 1.10)	1.08 (1.06, 1.10)	1.07 (1.05, 1.09)	1.06 (1.03, 1.08)	1.04 (1.01, 1.06)
Oil	1.06 (1.04, 1.08)	1.06 (1.04, 1.08)	1.06 (1.04, 1.08)	1.05 (1.03, 1.08)	1.04 (1.01, 1.07)
Soil	1.05 (1.04, 1.06)	1.04 (1.03, 1.06)	1.04 (1.02, 1.05)	1.04 (1.02, 1.06)	1.04 (1.02, 1.07)
Bio&Agr	1.07 (1.04, 1.09)	1.05 (1.02, 1.08)	1.02 (0.99, 1.06)	0.99 (0.95, 1.03)	0.97 (0.92, 1.01)
Industry	1.04 (1.02, 1.05)	1.03 (1.02, 1.04)	1.03 (1.01, 1.04)	1.02 (1.00, 1.04)	1.02 (1.00, 1.04)
$PM_{2.5}$ mass	1.12 (1.10, 1.14)	1.11 (1.08, 1.13)	1.07 (1.05, 1.10)	1.05 (1.02, 1.08)	1.03 (0.99, 1.06)

[a]Total number of observations = 323,782; person-years at risk = 6,317,235; number of deaths from natural mortality = 46,640.

[b]Follow-up period restricted to year 2000 and after, number of observations = 315,197 (97%); person-years at risk = 4,380,951 (69%); number of deaths from natural mortality = 39,321 (84%)

[c]Follow-up period restricted to year 2005 and after, number of observations = 303,022 (94%); person-years at risk = 2,905,753 (46%); number of deaths from natural mortality = 29,629 (64%)

[d]Follow-up period restricted to year 2008 and after, number of observations = 293,717 (91%); person-years at risk = 2,011,584 (32%); number of deaths from natural mortality = 21,785 (47%)

[e]Follow-up period restricted to year 2010 and after, number of observations = 284,325 (88%); person-years at risk = 1,434,077 (23%); number of deaths from natural mortality = 15,603 (33%)

Table D10. Associations between source-specific $PM_{2.5}$ and natural-cause mortality: applying source apportionment results from 5-fold robustness evaluation. HRs (95% CI) are presented for per 1 $\mu g/m^3$ increase in $PM_{2.5}$

	**Full**	**V1**	**V2**	**V3**	**V4**	**V5**
				**Single-source HR**		
Traffic	1.03 (1.02, 1.03)	1.03 (1.02, 1.03)	1.03 (1.02, 1.03)	1.03 (1.03, 1.04)	1.03 (1.02, 1.03)	1.03 (1.02, 1.03)
Oil	1.27 (1.19, 1.35)	1.22 (1.15, 1.29)	1.27 (1.19, 1.35)	1.37 (1.26, 1.50)	1.26 (1.18, 1.34)	1.29 (1.21, 1.38)
Soil	1.05 (1.04, 1.06)	1.05 (1.04, 1.06)	1.05 (1.03, 1.06)	1.04 (1.03, 1.05)	1.06 (1.04, 1.08)	1.06 (1.04, 1.07)
Bio&Agr	1.02 (1.01, 1.02)	1.02 (1.01, 1.02)	1.02 (1.01, 1.02)	1.02 (1.01, 1.02)	1.01 (1.01, 1.02)	1.01 (1.01, 1.02)
Industry	1.03 (1.02, 1.04)	1.03 (1.02, 1.05)	1.03 (1.02, 1.05)	1.03 (1.02, 1.04)	1.03 (1.02, 1.04)	1.03 (1.02, 1.04)
				**Multi-source HR**		
Traffic	1.02 (1.01, 1.03)	1.02 (1.01, 1.03)	1.02 (1.01, 1.02)	1.02 (1.02, 1.03)	1.02 (1.01, 1.03)	1.02 (1.01, 1.03)
Oil	1.13 (1.05, 1.22)	1.11 (1.04, 1.18)	1.13 (1.05, 1.22)	1.18 (1.07, 1.31)	1.12 (1.04, 1.20)	1.13 (1.05, 1.23)
Soil	1.01 (0.99, 1.03)	1.01 (0.99, 1.03)	1.01 (0.99, 1.03)	1.01 (0.99, 1.02)	1.01 (0.99, 1.03)	1.01 (0.99, 1.03)
Bio&Agr	1.01 (1.00, 1.02)	1.01 (1.00, 1.02)	1.01 (1.00, 1.02)	1.01 (1.00, 1.02)	1.01 (1.00, 1.01)	1.01 (1.00, 1.02)
Industry	1.00 (0.98, 1.01)	1.00 (0.98, 1.01)	1.00 (0.98, 1.02)	1.00 (0.98, 1.01)	1.00 (0.99, 1.01)	1.00 (0.99, 1.01)

Total number of observations= 323,782; person-years at risk =6,317,235; number of deaths from natural mortality = 46,640.

V1 to V5 are based on source apportionment analyses derived from evaluation datasets 1 to 5 (Appendix, Section C)

The main model adjusted for sub-cohort identification, age, sex, year of enrollment, smoking (status, duration, intensity, and $intensity^2$), BMI categories, marital status, employment status, and 2001 area-level mean income.

References

1. Thurston GD, Spengler JD. A quantitative assessment of source contributions to inhalable particulate matter pollution in metropolitan Boston. *Atmospheric Environment (1967)* 1985; **19**(1): 9-25.
2. Thurston GD, Ito K, Lall R. A Source Apportionment of U.S. Fine Particulate Matter Air Pollution. *Atmos Environ (1994)* 2011; **45**(24): 3924-36.
3. Hopke PK, Ito K, Mar T, Christensen WF, Eatough DJ, Henry RC, Kim E, Laden F, Lall R, Larson TV, Liu H, Neas L, Pinto J, Stölzel M, Suh H, Paatero P, Thurston GD. PM source apportionment and health effects: 1. Intercomparison of source apportionment results. *Journal of Exposure Science & Environmental Epidemiology* 2006; **16**(3): 275-86.
4. Hopke PK. Trace element concentrations in summer aerosols at rural sites in New York state and their possible sources and seasonal variations in the composition of ambient sulfatecontaining aerosols in the New York area. *Atmospheric Environment* 1982; **16**(5): 1279-80.
